# A framework for optimal control of oscillations and synchrony applied to non-linear models of neural population dynamics

**DOI:** 10.3389/fncom.2024.1483100

**Published:** 2024-12-06

**Authors:** Lena Salfenmoser, Klaus Obermayer

**Affiliations:** ^1^Institute of Software Engineering and Theoretical Computer Science, Technische Universitaet Berlin, Berlin, Germany; ^2^Bernstein Center for Computational Neuroscience Berlin, Berlin, Germany

**Keywords:** nonlinear optimal control, control of oscillations, control of synchrony, control of neural dynamics, neural population models

## Abstract

We adapt non-linear optimal control theory (OCT) to control oscillations and network synchrony and apply it to models of neural population dynamics. OCT is a mathematical framework to compute an efficient stimulation for dynamical systems. In its standard formulation, it requires a well-defined reference trajectory as target state. This requirement, however, may be overly restrictive for oscillatory targets, where the exact trajectory shape might not be relevant. To overcome this limitation, we introduce three alternative cost functionals to target oscillations and synchrony without specification of a reference trajectory. We successfully apply these cost functionals to single-node and network models of neural populations, in which each node is described by either the Wilson-Cowan model or a biophysically realistic high-dimensional mean-field model of exponential integrate-and-fire neurons. We compute efficient control strategies for four different control tasks. First, we drive oscillations from a stable stationary state at a particular frequency. Second, we switch between stationary and oscillatory stable states and find a translational invariance of the state-switching control signals. Third, we switch between in-phase and out-of-phase oscillations in a two-node network, where all cost functionals lead to identical OC signals in the minimum-energy limit. Finally, we (de-) synchronize an (a-) synchronously oscillating six-node network. In this setup, for the desynchronization task, we find very different control strategies for the three cost functionals. The suggested methods represent a toolbox that enables to include oscillatory phenomena into the framework of non-linear OCT without specification of an exact reference trajectory. However, task-specific adjustments of the optimization parameters have to be performed to obtain informative results.

## 1 Introduction

OCT is a mathematical framework that offers methods to compute efficient stimulation for linear or non-linear dynamical systems (Kirk, 2004) with numerous applications in science, engineering, and operations research. With OCT, a stimulating signal can be designed such that the dynamical system under consideration behaves in a desired manner. Conventionally, a reference trajectory is defined that the state variable of the dynamical system should mimic. A cost functional trades the closeness between the actual and a reference state (typically, the squared difference between the reference and target states integrated over time) against the input strength of the control (typically, the squared control input integrated over time). The optimal control (OC) minimizes the cost functional.

Control of neural system has been studied intensively both theoretically and experimentally (Kao and Hennequin, 2019; Suppa et al., 2016; Liu et al., 2018). Closed-loop control and machine learning approaches have been applied, for example, to modulate brain activity (Grosenick et al., 2015; Tafazoli et al., 2020; Park et al., 2019) and optimize stimulation protocols for the treatment of neurological disorders (Yu et al., 2020; Chandrabhatla et al., 2023). Open-loop optimal control has been computed for non-linear models of neural population dynamics (Salfenmoser and Obermayer, 2022, 2023) and networks thereof Chouzouris et al. (2021). Beyond that, network control theory has widely been applied to linear models of brain networks to understand how network structure determines the role of brain circuits to control network function (McGowan et al., 2022), or how altered structural connectivity is linked to mental disorders (Zöller et al., 2021).

In neural systems, oscillatory phenomena commonly emerge and spread over various spatial and temporal scales. To control neural oscillations, OCT has mostly been applied to phase-reduced models of oscillatory systems (e.g., Dasanayake and Li, 2011; Zlotnik and Li, 2012; Pyragas et al., 2020; Wilson and Moehlis, 2014). Phase reduction techniques enable to drastically simplify complex dynamical system evolving along a stable limit cycle by parametrizing their dynamics via their phase only (Pietras and Daffertshofer, 2019). As the oscillation phase becomes the (only) state variable, synchronization directly translates to phase alignment, and optimal synchronization and desynchronization can be studied straightforwardly by conventional OCT methods. The low computational complexity of phase-reduced models enables OC studies of large-scale networks (e.g., Bomela et al., 2023). However, phase-reduced models can by construction only describe trajectories along the stable limit cycles of coupled oscillators. Control strategies are limited to phase shifts along these limit cycles and cannot capture larger deviations from it. The phase-reduced approximation breaks down at a bifurcation, and the model dynamics may not be described accurately close to a bifurcation. Beyond phase model studies, delayed feedback control (Popovych et al., 2006; Hövel et al., 2010) and model-free machine learning methods (Krylov et al., 2020; Vu et al., 2024) have been applied to discuss optimal synchronization or desynchronization in multi-dimensional models of neural activity.

There are different classes of methods to compute OC for non-linear systems (Rao, 2010). Model-based approaches use the mathematical description of a dynamical system: While indirect methods reformulate the optimization problem such that optimality conditions are obtained, from which the optimal control can be computed, direct methods discretize the continuous control problem such that it becomes an optimization problem in finite space. Model-free approaches learn control strategies from a sufficient amount of data of how the system behaves under stimulation, without using any information on the analytical model description (Koryakovskiy et al., 2017).

In this study, we compute OC using the indirect adjoint method, which enables to formulate an expression for the gradient of the cost functional with respect to the control. It can be applied to any linear or non-linear dynamical system and is, in principle, analytic. However, the arising equations cannot be solved analytically in most non-linear systems and require numerical solutions. Up to the uncertainties of the numerical computation, the method remains exact. Control shapes are entirely determined by the system dynamics at any point in the state space and for any dynamical regime. The method is applicable to arbitrary networks; however, in practice, computational complexity will limit network sizes.

Standard mathematical methods borrowed from OCT are limited in their applicability to oscillatory phenomena in (unreduced) complex dynamical systems: The specification of a reference trajectory is overly restrictive since the exact shape of the oscillatory trajectory, including properties such as amplitude and phase, may often be irrelevant. We therefore adapt the OCT framework and suggest to replace the cost functional that measures closeness between actual and reference state by one of three alternative cost functionals:

- The *Fourier cost* evaluates the Fourier component corresponding to a target oscillation frequency.
- The *cross-correlation cost* evaluates the pairwise cross-correlation of the dynamical state of all nodes in a network (Chouzouris et al., 2021).
- The *variance cost* evaluates the variance of the activity throughout a network.

The Fourier cost enables new applications of OCT to induce oscillations when the system is in a fixed point without specifying a reference trajectory. All three cost functionals are evaluated and compared for switching between stationary states and for the control of network (de-) synchronization. The variance cost is inspired by studies on the control of frequency synchronization in phase oscillator models (e.g., Taher et al. (2019)), and a similar cost functional has recently been studied using a data-driven approach (see Vu et al., 2024). The cross-correlation cost has been studied before in the context of network synchronization (cf. Chouzouris et al., 2021) and is included for comparison.

We demonstrate how to include these cost functionals into the adjoint method, such that all benefits from this method are inherited. This enables to study efficient control strategies in complex systems for a repertoire of control tasks that cannot be captured by phase-reduced oscillator models. Potential applications go beyond (de-) synchronization of oscillating networks and may combine oscillatory and non-oscillatory states, e.g., in multistable systems, or across bifurcation boundaries.

In this study, we use two non-linear models of neural populations dynamics as example systems. We study the Wilson-Cowan (WC) model (Wilson and Cowan, 1972, 1973), a simple, phenomenological neural mass model, and the mean-field EIF model, a biophysically motivated mean-field model of excitatory and inhibitory exponential integrate-and-fire (EIF) neurons (Augustin et al., 2017; Cakan and Obermayer, 2020). The mean-field EIF model exhibits more dynamical variety and hence enables to study control tasks that are not accessible with the WC model only. In these models, the OC cannot be computed analytically. Instead, the cost gradient is computed numerically, and the OC is approached by gradient descent.

This article is structured as follows: Section 2.1 provides an introduction to OCT, and Section 2.2 presents our adjustments to target the problem of controlling oscillations and synchrony. In Section 2.3, we introduce the two models of neural population dynamics that serve as example systems in this study. Section 2.4 presents the implementation of our OC methods, and we provide details on the numerical simulations and on the convergence properties of the gradient descent procedure. In Section 3, we present our results. We first study the efficacy of the Fourier cost to enforce oscillations from stationary states in a single-node system (see Sections 3.1 and 3.2.1). Then, we apply the Fourier cost, the cross-correlation cost, and the variance cost to switch between oscillatory states (see Section 3.2.2). Finally, we synchronize or desynchronize oscillating networks with the three proposed cost functionals (see Section 3.3). In Section 4, we summarize our findings, discuss the limitations of our method, and give an outlook on potential future areas of application.

## 2 Methods

### 2.1 Optimal control of non-linear dynamical systems

We study controlled non-linear dynamical systems that may be defined by ordinary differential equations (ODEs) or differential-algebraic equations (DAEs) and may include delays. We denote such systems by


(1) $$\begin{array}{l} \vec{h}\left(\vec{x}\left(t\right),\dot{\vec{x}}\left(t\right),\vec{x}\left(t-d_{1}\right),\vec{x}\left(t-d_{2}\right),...,\vec{x}\left(t-d_{N_{d}}\right),\vec{u}\left(t\right)\right)=0, \end{array}$$

with the state vector $\vec{x}$, its time derivative $\dot{\vec{x}}$, and *N*_*d*_ potentially different delays *d*_*i*_. In this study, we consider network systems with *N* network nodes and a finite simulation time. The state vector $\vec{x}$ contains the stacked state vectors ${\vec{x}}_{n},n\in \left\{1,2,...N\right\}$, of all nodes. Similarly, $\vec{h}$ contains the stacked system dynamics of all network nodes. We denote the dimensionality of the state vector of a single node as *N*_*x*_, such that $\left\{\vec{x},\vec{h}\right\}\in {\mathbb{R}}^{N\cdot N_{x}}$. $\vec{u}\left(t\right)$ is the control input, which may effect one or more components of ${\vec{x}}_{n}$ for each node in a network. The OC formalism provides methods to compute a control $\vec{u}\left(t\right)$ that is most efficient for a particular purpose. A cost functional *F* measures the efficiency of a control. It is conventionally defined as


(2) $$\begin{array}{r} F=w_{\text{P}}\underset{=F_{\text{P}}}{\underset{︸}{\frac{1}{2\left(T-t_{0}\right)}\int_{t_{0}}^{T}{\left(\vec{x}\left(t\right)-\tilde{x}\left(t\right)\right)}^{2}dt}}+\underset{=F_{\text{E}}}{\underset{︸}{\frac{1}{2}\int_{0}^{T}{\vec{u}}^{2}\left(t\right)dt}}\\ =\int_{0}^{T}f\left(\vec{x},\vec{u}\right)dt. \end{array}$$

The first term *F*_P_ measures the closeness of the trajectory of the state vector $\vec{x}\left(t\right)$ to a given target trajectory of state vectors $\tilde{x}\left(t\right)\in {\mathbb{R}}^{N\cdot N_{x}}$ and is referred to as the precision cost. The second term *F*_E_ measures the strength of the control signal and is referred to as the energy cost. *T* denotes the considered time interval. Inaccuracy is penalized for *t*_0_ ≤ *t* ≤ *T*, while control strength is penalized for the complete time interval. When controlling a switch between two stable states, we set *t*_0_>0 to account for a finite transition time. The OC ${\vec{u}}^{*}$ minimizes the cost,


(3) $$\begin{array}{l} {\vec{u}}^{*}=\arg\underset{\vec{u}}{\min}F. \end{array}$$

The weight *w*_P_ can be tuned to trade precision against the required control strength.

In the following Section 2.2, we will consider new cost functionals $F_{\text{X}}\left(\vec{x}\right)$ which go beyond the precision cost $F_{\text{P}}\left(\vec{x}\right)$ defined in Equation 2, i.e., $F_{\text{P}}\left(\vec{x}\right)\to F_{\text{X}}\left(\vec{x}\right)$. To be able to apply the *adjoint method*, we require that the derivative of the cost functional with respect to the control can be written in the form


(4) $$\begin{array}{l} \frac{dF_{\text{X}}\left(\vec{x}\right)}{d\vec{u}}=\int_{0}^{T}\vec{g}\left(\vec{x}\right)\frac{d\vec{x}}{d\vec{u}}dt, \end{array}$$

where $\vec{g}\left(\vec{x}\right)$ is some function of the state variable $\vec{x}$ (see Supplementary material).

The adjoint method (Kirk, 2004) enables to compute the derivative $\frac{d}{d\vec{u}}F$ of the cost functional with respect to the control. The adjoint state $\vec{\lambda }\in {\mathbb{R}}^{N\cdot N_{x}}$ is defined by the differential equation


(5) $$\begin{array}{r} \dot{\vec{\lambda }}{\left(t\right)}^{T}\frac{\partial \vec{h}\left(t\right)}{\partial \dot{\vec{x}}}=\vec{g}\left(\vec{x}\right)+{\vec{\lambda }}^{T}\left(t\right)\frac{\partial \vec{h}\left(t\right)}{\partial \vec{x}}\\ +\overset{N_{d}}{\underset{i=1}{\sum }}\chi_{\left[0,T-d_{i}\right]}{\vec{\lambda }}^{T}\left(t+d_{i}\right)\frac{\partial \vec{h}\left(t+d_{i}\right)}{\partial \vec{x}}-{\vec{\lambda }}^{T}\frac{d}{dt}\frac{\partial \vec{h}}{\partial \dot{\vec{x}}} \end{array}$$

with the final condition $\vec{\lambda }\left(T\right)=0$. χ_[_*t*__*a*_, *t*_*b*_]_ denotes the indicator function of the time interval [*t*_*a*_, *t*_*b*_]. Above expression contains vectors $\left\{\vec{\lambda },\dot{\vec{\lambda }},\vec{g}\left(\vec{x}\right)\right\}\in {\mathbb{R}}^{N\cdot N_{x}}$, and matrices $\left\{\frac{\partial \vec{h}}{\partial \dot{\vec{x}}},\frac{\partial \vec{h}}{\partial \vec{x}}\right\}\in {\mathbb{R}}^{\left(N\cdot N_{x}\right)\times \left(N\cdot N_{x}\right)}$, with ${\left(\frac{\partial \vec{h}}{\partial \dot{\vec{x}}}\right)}_{ij}=\frac{\partial h_{i}}{\partial {ẋ}_{j}}$ (indices *i* and *j* denote the *i*th and *j*th vector components). Note that for the dynamical systems studied in this work, $\frac{\partial \vec{h}}{\partial \dot{\vec{x}}}$ is a constant such that the last term in Equation 5 vanishes. The adjoint state $\vec{\lambda }$ is computed by backward integration. It enables to compute the cost derivative


(6) $$\begin{array}{l} \frac{d}{d\vec{u}}F=\int_{0}^{T}\frac{\partial f}{\partial \vec{u}}+{\vec{\lambda }}^{T}\frac{\partial \vec{h}}{\partial \vec{u}}dt. \end{array}$$

The integrand $\frac{\partial f}{\partial \vec{u}}+{\vec{\lambda }}^{T}\frac{\partial \vec{h}}{\partial \vec{u}}$ is the cost gradient as a function of time, which is required for the gradient descent. A derivation of the adjoint method is provided in the Supplementary Section 1.

When studying oscillatory phenomena and the control thereof, a well-defined target trajectory ${\tilde{x}}_{n}\left(t\right)$ for each network node implies that the exact shape of the oscillation, including amplitude and phase, is set. This might be overly restrictive in certain scenarios. In the next section, we will, therefore, introduce alternative cost functionals to replace the precision cost *F*_P_ in Equation 2.

### 2.2 Optimal control of oscillations and synchrony

For the cost functionals that measure oscillation and synchronization, we consider only one relevant state variable for each network node. The formalism we present is, however, not generally limited to this simplification and can be extended straightforwardly. We drop the vector arrow and denote the observable component of the state variable by *x*_*n*_(*t*) for network node *n*.

To enforce phase and frequency synchronization in a network at frequency $\tilde{f}$, we replace *F*_P_ in Equation 2 by the *synchronization Fourier cost*,


(7) $$F_{\text{F}}^{\text{sync}}=-\frac{1}{N^{2}{\left(T-t_{0}\right)}^{2}}|\int_{t_{0}}^{T}\Sigma_{x}(t)\cdot e^{-i\omega t}\;dt{|}^{2}$$

with $\Sigma_{x}\left(t\right)=\overset{N}{\underset{n=1}{\sum }}x_{n}\left(t\right)$ and $\omega =2\pi \tilde{f}$. $F_{\text{F}}^{\text{sync}}$ is the squared Fourier component corresponding to the frequency $\tilde{f}$ of the sum of observables over all *N* network nodes. The computation of the adjoint state using Equation 5 requires


(8) $$\begin{array}{l} {\left(g_{\text{F}}^{\text{sync}}\left(\vec{x}\right)\right)}_{k}=-\frac{2}{N^{2}{\left(T-t_{0}\right)}^{2}}\int_{t_{0}}^{T}\Sigma_{x}\left(\tau \right)\cdot \cos\left(\omega \left(t-\tau \right)\right)d\tau . \end{array}$$

The derivation of Equation 8 is provided in the Supplementary Section 2.1.

To enforce frequency synchronization in a network at frequency $\tilde{f}$ irrespective of the relative phases of its nodes, we replace *F*_P_ in Equation 2 by the *oscillation Fourier cost*


(9) $$F_{\text{F}}^{\text{osc}}=-\frac{1}{N{\left(T-t_{0}\right)}^{2}}\sum_{n=1}^{N}|\int_{t_{0}}^{T}x_{n}(t)\cdot e^{-i\omega t}\;dt{|}^{2}.$$

$F_{\text{F}}^{\text{osc}}$ is the sum over all *N* network nodes of the node-wise squared Fourier component corresponding to the frequency $\tilde{f}$. The computation of the adjoint state using Equation 5 requires


(10) $$\begin{array}{l} {\left(g_{\text{F}}^{\text{osc}}\left(\vec{x}\right)\right)}_{k}=-\frac{2}{N{\left(T-t_{0}\right)}^{2}}\int_{t_{0}}^{T}x_{k}\left(\tau \right)\cdot \cos\left(\omega \left(t-\tau \right)\right)d\tau . \end{array}$$

The derivation of Equation 10 is provided in the Supplementary Section 2.2. For *N* = 1, synchronization and oscillation Fourier costs are identical and enforce oscillations at frequency $\tilde{f}$. We will denote the cost by *F*_F_ in this case. The oscillation Fourier cost $F_{\text{F}}^{\text{osc}}$ improves, if the power corresponding to the target frequency $\tilde{f}$ increases in any network node, irrespective of the relative phases. The synchronization Fourier cost $F_{\text{F}}^{\text{sync}}$ improves, either if oscillatory network activity at frequency $\tilde{f}$ synchronizes, or if the power in the network nodes corresponding to $\tilde{f}$ increases^1^.

To enforce phase synchronization in a network irrespective of the oscillation frequency, we replace *F*_P_ in Equation 2 by either the cross-correlation cost or the variance cost. The cross-correlation cost (Chouzouris et al., 2021) is defined as


(11) $$\begin{array}{l} F_{\text{cc}}=-\frac{2}{N\left(N-1\right)\left(T-t_{0}\right)}\int_{t_{0}}^{T}\overset{N}{\underset{\begin{array}{c} n=1\\ l=n+1 \end{array}}{\sum }}\frac{\left(x_{n}\left(t\right)-{\bar{x}}_{n}\right)\left(x_{l}\left(t\right)-{\bar{x}}_{l}\right)}{\sigma \left(x_{n}\right)\sigma \left(x_{l}\right)}dt, \end{array}$$

where $\bar{x}=\frac{1}{T-t_{0}}\int_{t_{0}}^{T}x\left(t\right)dt$ is the temporal mean of *x*(*t*), and $\sigma^{2}\left(x\right)=\frac{1}{T-t_{0}}\int_{t_{0}}^{T}{\left(x\left(t\right)-\bar{x}\right)}^{2}dt$ is its variance. The computation of the adjoint state using Equation 5 requires


(12) $$\begin{array}{r} {\left(g_{\text{cc}}(\vec{x})\right)}_{k}=\frac{2}{N(N-1)(T-t_{0})}\sum_{\begin{array}{c} l=1\\ l\neq k \end{array}}^{N}\frac{\left(x_{l}(t)-{\bar{x}}_{l}\right)}{\sigma (x_{k})\sigma (x_{l})}\\ -\frac{(x_{k}(t)-{\bar{x}}_{k}){ℐ}_{kl}}{\left(T-t_{0})\sigma {\left(x_{k}\right)}^{3}\sigma (x_{l}\right)}, \end{array}$$

with $I_{kl}=\int_{t_{0}}^{T}\left(x_{k}\left(t\right)-{\bar{x}}_{k}\right)\left(x_{l}\left(t\right)-{\bar{x}}_{l}\right)dt$. The derivation of Equation 12 is provided in the Supplementary Section 2.3.

The variance cost is defined as


(13) $$\begin{array}{l} F_{\text{var}}=\frac{1}{N\left(T-t_{0}\right)}\int_{t_{0}}^{T}\overset{N}{\underset{n=1}{\sum }}{\left(x_{n}\left(t\right)-\bar{x}\left(t\right)\right)}^{2}dt, \end{array}$$

where $\bar{x}\left(t\right)=\frac{1}{N}\overset{N}{\underset{n=1}{\sum }}x_{n}\left(t\right)$ is the network mean of *x*(*t*). The computation of the adjoint state using Equation 5 requires


(14) $$\begin{array}{l} {\left(g_{\text{var}}\left(\vec{x}\right)\right)}_{k}=\frac{2\left(x_{k}\left(t\right)-\bar{x}\left(t\right)\right)}{N\left(T-t_{0}\right)}. \end{array}$$

The derivation of Equation 14 is provided in the Supplementary Section 2.4.

Both *F*_cc_ and *F*_var_ are defined for networks with *N*≥2 and cannot be applied to a single-node system.

#### 2.2.1 Control weights

When replacing *F*_P_ in Equation 2 by one of the abovementioned cost functionals, we also introduce the respective weights, $w_{\text{F}}^{\text{sync}}$, $w_{\text{F}}^{\text{osc}}$, *w*_cc_, or *w*_var_ replacing *w*_P_ in Equation 2. By using a negative weight, we can suppress a specific oscillation mode (oscillation Fourier cost) and enforce desynchronization instead of synchronization (synchronization Fourier cost), enforce a small cross-correlation, or enforce a large variance (e.g., in the case of a desynchronization task).

### 2.3 Models of neural population dynamics

We apply our methods to two models of neural population dynamics, the WC model (see Section 2.3.1) and the mean-field EIF model (see Section 2.3.2). The former is a simple, two-dimensional system of ordinary differential equations (ODEs), while the latter is a high-dimensional system of delay differential-algebraic equations (DDAEs).

#### 2.3.1 The Wilson-Cowan model

The WC model describes the activity of coupled excitatory and inhibitory neural populations (Wilson and Cowan, 1972, 1973). The dynamics are defined by


(15) $$\begin{array}{l} \tau_{E}{\dot{E}}_{n}(t)=-E_{n}(t)+(1-E_{n}(t))\cdot S(c_{EE}E_{n}(t)\\ -c_{EI}I_{n}(t)+E^{\text{ext}}+u_{n}(t)+c_{\text{gl}}\sum_{m=1}^{N}C_{nm}E_{m}(t-D_{nm}))\\ \tau_{I}{\dot{I}}_{n}(t)=-I_{n}(t)+(1-I_{n}(t))\cdot S(c_{IE}E_{n}(t)-c_{II}I_{n}(t)+I^{\text{ext}}). \end{array}$$

**Figure 1:**
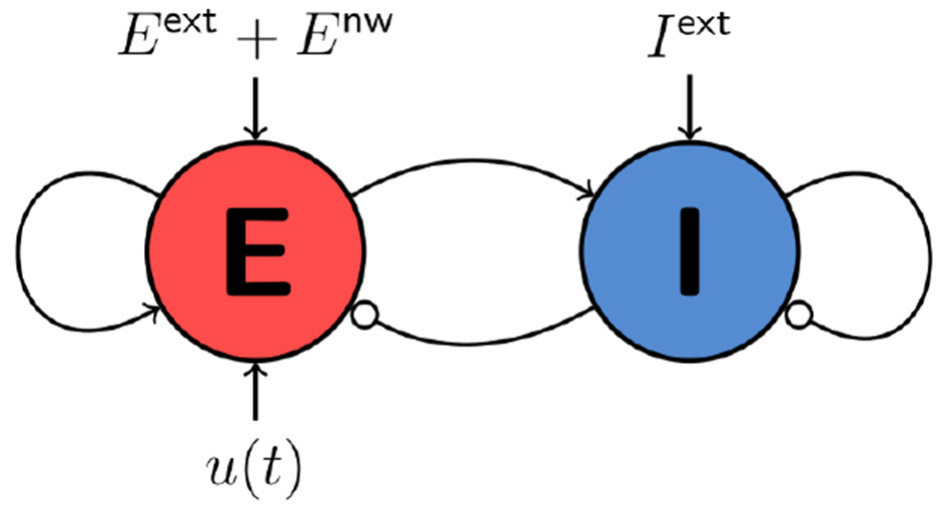
Cartoon of a single node of the WC network model. Static external inputs are indicated by *E*^ext^ and *I*^ext^, network inputs by *E*^nw^, and control inputs by *u*(*t*).

The activity variables *E*_*n*_(*t*) and *I*_*n*_(*t*) denote the fraction of excitatory and inhibitory neurons that are active at time *t* in node *n* in a network of *N* nodes. τ_*E*_ and τ_*I*_ are the decay time constants of the excitatory and inhibitory activity, respectively. *c*_βα_ denotes the coupling from population α to population β with α, β∈{*E, I*}. *E*^ext^ and *I*^ext^ are static external inputs that serve as order parameters for the network. *u*_*n*_(*t*) is the time-dependent control, *c*_gl_ denotes the global coupling strength, and *C* and *D* are the *N*×*N* coupling and delay matrices. The transfer function *S*(*x*) = (1+exp−γ(*x*−μ))^−1^ computes a synaptic input to *E*_*n*_ or *I*_*n*_ from the sum of all inputs. Numerical values for the parameters are given in Table 1. Figure 1 sketches the dynamical interactions between the excitatory and inhibitory populations of a single WC node in a network. Excitatory and inhibitory population are recurrently coupled. Only the excitatory population receives network and control inputs.

**Table 1:** Parameters of the WC model.

**Parameter**	**Explanation**	**Value**
τ_*E*_	decay constant of *E*	2.5
τ_*I*_	decay constant of *I*	3.75
γ	gain	1.5
μ	firing threshold	3.0
*c*_*EE*_	E to E coupling	16
*c*_*EI*_	I to E coupling	12
*c*_*IE*_	E to I coupling	15
*c*_*II*_	I to I coupling	3

**Figure 2:**
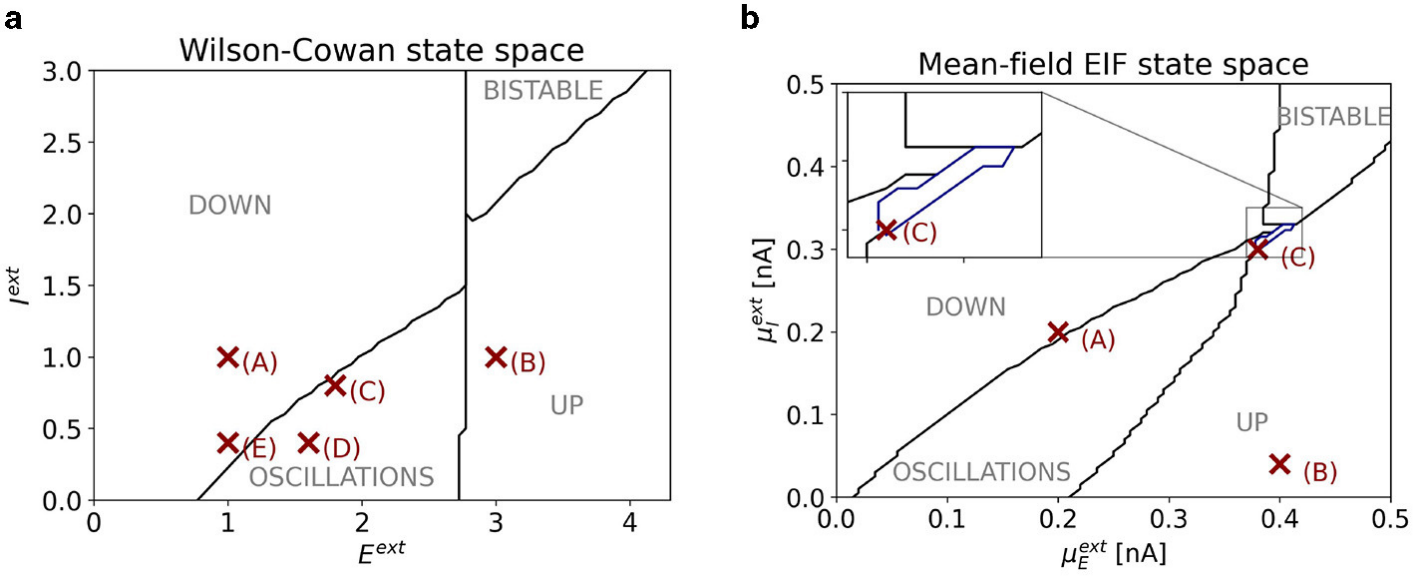
**(A)** Slice of state space of one node of the WC model. The horizontal (vertical) axis corresponds to the external input *E*^ext^ (*I*^ext^). The four dynamical regimes are separated by black lines. Red markers indicate the points in state space at which control tasks are studied. Their coordinates are (1, 1) for point (A); (3, 1) for point (B); (1.8, 0.8) for point (C); (1.6, 0.4) for point (D); (1, 0.4) for point (E). **(B)** Slice of state space of one node of the mean-field EIF model. The horizontal (vertical) axis corresponds to the external input $\mu_{E}^{\text{ext}}$ ($\mu_{I}^{\text{ext}}$). The five dynamical regimes are separated by black and blue lines. In the inset, we zoom into the regime bounded by the blue line, where stable oscillations coexist with a stable up state. Red markers indicate the points in state space at which control tasks are studied. Their coordinates are (0.2 nA, 0.2 nA) for point (A); (0.4 nA, 0.04 nA) for point (B); (0.38 nA, 0.3 nA) for point (C).

Figure 2A shows a slice of the state space of the one-node WC model with parameters as given in Table 1. Depending on the external inputs *E*^ext^ and *I*^ext^, the system exhibits a state of constant low activity (“down state”), a state of constant high activity (“up state”), an oscillatory state, or a bistable state, where stable states of constant low and high activity coexist. Points (A)–(E) mark locations in state space, for which control tasks are studied (see Section 3).

#### 2.3.2 The mean-field EIF model

The mean-field EIF model (Augustin et al., 2017; Cakan and Obermayer, 2020) captures the dynamics of a network of randomly and sparsely connected exponential excitatory and inhibitory integrate-and-fire neurons (Brette and Gerstner, 2005) in the limit of infinitely many neurons. In this limit, a dimensionality reduction can be performed, and the mean firing rates *r*_*E*_ and *r*_*I*_ of the excitatory and inhibitory populations can be obtained as the activity state variables. *r*_*E*_ and *r*_*I*_ are functions of the mean membrane currents and their variances. The model dynamics are described by a 16-dimensional system of delay differential-algebraic equations (DDAEs), which is provided in the Supplementary Section 3. Schematically, the dynamical interactions of the mean-field EIF model can be represented similarly as shown in Figure 1. Note that external inputs are denoted differently as $\mu_{E}^{\text{ext}}$ and $\mu_{I}^{\text{ext}}$.

Figure 2B shows a slice of state space of the one-node mean-field EIF model. Depending on the external inputs $\mu_{E}^{\text{ext}}$ and $\mu_{I}^{\text{ext}}$, the system exhibits a state of constant low activity (“down state”), a state of constant high activity (“up state”), an oscillatory state, or a bistable state, where stable states of constant low and high activity coexist. Beyond that, there is a small region between the oscillatory and bistable regimes where we find bistability between an oscillatory and a down state (marked in blue). Points (A)–(C) mark locations in state space, for which control tasks are studied (see Section 3).

#### 2.3.3 Parameters for network control

We study two control tasks for a WC network with nodes coupled via the excitatory population. In a preliminary state-space exploration for various combinations of network and coupling parameters, we hand-picked adequate parameters such that the desired control tasks can be studied.

For the two-node network studied in Section 3.2.2, coupling and delay matrices are given by


(16) $$\begin{array}{l} C=\left(\begin{array}{cc} 0 & 1\\ 1 & 0 \end{array}\right),\;D=\left(\begin{array}{cc} 0 & 9.5\\ 9.5 & 0 \end{array}\right). \end{array}$$

**Figure 3:**
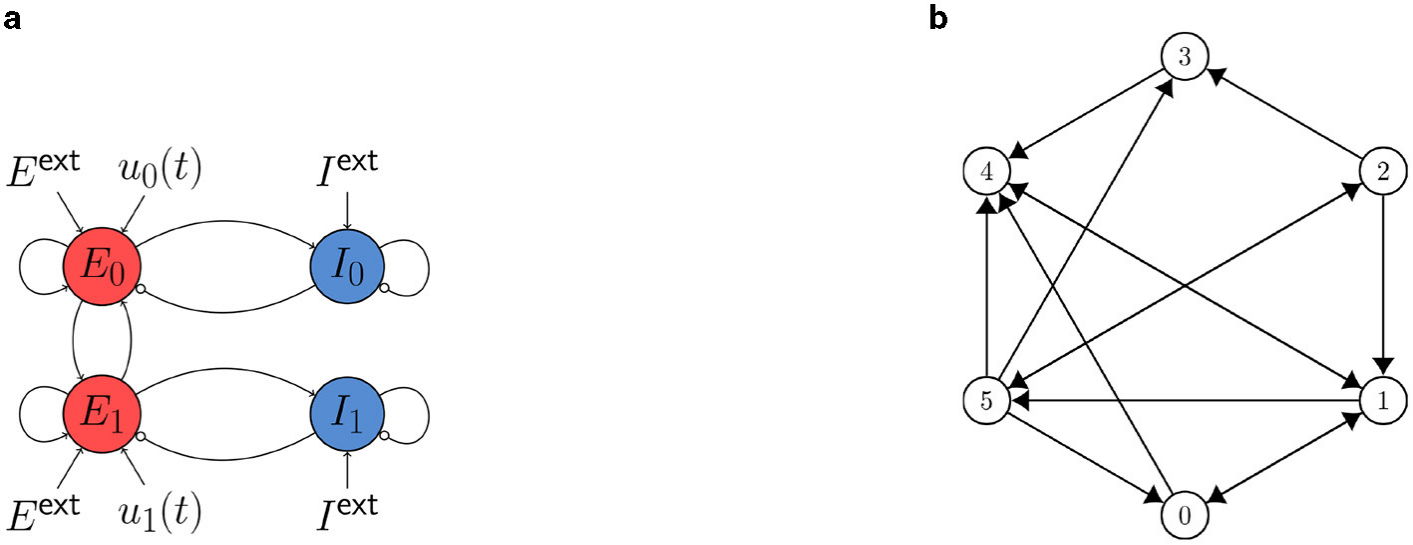
**(A)** Cartoon of the neural mass model with two WC nodes studied in Section 3.2.2. Static external inputs are indicated by *E*^ext^ and *I*^ext^ and control inputs by *u*_0,1_ (*t*). The nodes are coupled via the excitatory population with a global coupling strength *c*_gl_. **(B)** Six-node system studied in Section 3.3.

The coupling scheme is sketched in Figure 3A. We set *c*_gl_ = 1.8 (cf. Equation 15) and find a bistable state at point (C) (see Figure 2A), where a stable in-phase oscillation (IP) with period $f_{\text{IP}}^{-1}=13.89$ coexists with a stable out-of-phase oscillation (OOP) with period $f_{\text{OOP}}^{-1}=22.72$.

For the six-node network studied in Section 3.3, coupling and delay matrices are given by


(17) $$\begin{array}{l} C=\left(\begin{array}{cccccc} 0 & 1 & 0 & 0 & 0 & 1\\ 1 & 0 & 1 & 0 & 1 & 0\\ 0 & 0 & 0 & 0 & 0 & 1\\ 0 & 0 & 1 & 0 & 0 & 1\\ 1 & 1 & 0 & 1 & 0 & 1\\ 0 & 1 & 1 & 0 & 0 & 0 \end{array}\right),\;D=\left(\begin{array}{cccccc} 0 & 12 & 0 & 0 & 0 & 8\\ 8 & 0 & 13 & 0 & 1 & 0\\ 0 & 0 & 0 & 0 & 0 & 9\\ 0 & 0 & 4 & 0 & 0 & 11\\ 5 & 17 & 0 & 14 & 0 & 18\\ 0 & 0 & 3 & 0 & 0 & 0 \end{array}\right). \end{array}$$

The coupling scheme is sketched in Figure 3B. We set *c*_gl_ = 0.8 (cf. Equation 15) and find a state of asynchronous oscillation at point (D) and a state of synchronous oscillation at point (E) (see Figure 2A).

Note that the state-space diagram in Figure 2A only shows the bifurcations boundaries of the one-node system. Bifurcation boundaries change depending on the choice of network and coupling parameters.

Parameters were chosen such that relevant control problems could be defined for evaluating the proposed cost functionals. Note, however, that the presented method can be applied to any choice of coupling and delay matrices.

### 2.4 Implementation and numerical simulations

#### 2.4.1 Open-source implementation

Numerical computations are based on the neurolib simulation framework (Cakan et al., 2023). neurolib is an open-source library that contains several models of neural population dynamics and enables to combine them to network models of arbitrary size and structure. Beyond that, methods to compute OC in its standard formulation (see Section 2.1) are included. In this study, we expand neurolib by an extension that enables to compute OC signals for oscillation and synchronization tasks. This extension is openly available on GitHub at https://github.com/lenasal/neurolib/tree/OC_osc_sync.

#### 2.4.2 Simulation accuracy

All simulations in this study have a duration of several hundred time units (dimensionless in the WC model, *ms* in the mean-field EIF model). We chose an integration step size *dt* = 0.1 (WC) or *dt* = *0.1ms* (mean-field EIF) and validated that there are no qualitative differences if a smaller integration time step is used.

#### 2.4.3 Fourier cost in limited-time simulations

When evaluating the Fourier spectrum of a trajectory obtained in a limited-time simulation, the spectral peaks are the sharper, the longer the simulation duration. If, however, the simulation time is relatively short (as in the control tasks considered in this study), the Fourier spectrum of any oscillation shows broad peaks (see Supplementary Figure S1). For tasks, in which we want to switch to a specific oscillatory state (Section 3.2), this leads to a tolerance against variations in the frequency $\tilde{f}$ compared to the natural frequency of the target state.

#### 2.4.4 Initialization

As an initial control guess, we use *u*_0_ = 0 in most tasks investigated in Section 3, with two exceptions.

First, in Section 3.1, we control the mean-field EIF model in its stationary down state. In this state, we encounter numerical problems when the gradient of the cost functional $\frac{\partial f}{\partial \vec{u}}$ is computed. This is due to the fact that the activity, the excitatory firing rate *r*_*E*_(*t*), is not given in terms of an analytical function, but in terms of a transfer function, for which no analytical closed-form expression exists (for details on the mathematical model, see also Supplementary Section 3). In simulations, *r*_*E*_(*t*) is, therefore, interpolated using a pre-computed table. In the down state, gradients almost vanish, preventing an effective OC computation if the control is not initialized reasonably. Hence, we initialize the OC algorithm with a sinusoidal initial control *u*_0_ which oscillates at the target frequency $\tilde{f}$.

Second, in Section 3.2.2, we study a control task where all network nodes exhibit the exact same time evolution in the uncontrolled, initial state. When applying the variance cost, both cost and cost gradient vanish (see Equations 13 and 14), and the gradient descent cannot be performed. To circumvent this problem, we initialize the OC algorithm with an initial control that randomly fluctuates around zero with a small amplitude.

#### 2.4.5 Choice of weights

The numerical values of the weights *w*_F_, *w*_cc_, or *w*_var_ determine how accuracy and control strength are traded against each other. On the one hand, when choosing the weight below a certain threshold value, which is individual for each cost functional and task, the OC equals zero, ${\vec{u}}^{*}\left(t\right)=0$. Any finite input would increase the total cost via *F*_E_ more than it might improve *F*_F_, *F*_cc_, or *F*_var_. This threshold value is a lower limit to the choice of weights. On the other hand, when choosing a very large weight, the algorithm will return very strong control signals, and the controlled activity will reach its upper limits determined by the system dynamics. Physically, such results might be difficult to interpret. Computationally, the numerical integration might fail when the activity variable is continuously pushed against its upper bound. Hence, too large weight values must be avoided.

We study two different types of control tasks: First, there are control tasks that force a system to behave in a way that cannot be maintained naturally (driving oscillations from a stationary state, Sections 3.1, and synchronization (desynchronization) of desynchronized (synchronized) oscillations, Section 3.3). For these tasks, the weights are chosen after preliminary investigations such that control signals are reasonably strong. Second, there are control tasks in which we initiate a switch between two stable states (Section 3.2). For these tasks, we want to find the minimum-energy transition. Therefore, we dynamically adjust the weights, starting with a relatively large value of *w*_F_, *w*_cc_, or *w*_var_, and decrease the respective weight every few (hundred) iterations until convergence, such that low-energy transitions are enforced. For these cases, we only provide the initial and the final numerical weight value.

#### 2.4.6 Measurement interval and control interval

In our simulations, we evaluate $F_{\text{F}}^{\text{sync}}$, $F_{\text{F}}^{\text{osc}}$, *F*_cc_, or *F*_var_ in the time interval [*t*_0_, *T*] (“measurement interval”). Similarly, we enable control only in a limited time interval, which we denote by $\left[t_{0}^{\text{C}},t_{1}^{\text{C}}\right]$ (“control interval”), i.e., *u*(*t*)≠0 only for $t\in \left[t_{0}^{\text{C}},t_{1}^{\text{C}}\right]$. Naturally, we chose $t_{0}\geq t_{0}^{\text{C}}$.

For control tasks that force a system to behave in way that cannot be maintained without control input (driving oscillations from a stationary state, Section 3.1, and synchronization (desynchronization) of desynchronized (synchronized) oscillations, Section 3.3), we set $t_{0}=t_{0}^{\text{C}}$ and $T=t_{1}^{\text{C}}$. For such tasks, no sustainable, long-lasting effect can be achieved, and we only consider the interval in which control is active.

For control tasks that initiate a switch between two stable states (Section 3.2), the algorithm might fail if there is an overlap of measurement interval and control interval as we might encounter a (local) optimum at which the control reduces the cost for $t\in \left[t_{0},t_{1}^{\text{C}}\right]$ without initiating the intended state switch. On the other hand, the gradient computation with the adjoint method becomes less precise if there is no overlap between measurement interval and control interval. Preliminary simulations help to determine well-suited values for $t_{0}^{\text{C}},t_{1}^{\text{C}},t_{0}$, and *T*. We chose a setup with no overlap between measurement interval and control interval for single-node systems (Section 3.2.1), and a setup with overlap for multi-node systems (Section 3.2.2).

#### 2.4.7 Local optima

We use gradient descent to reach a minimum of the cost functional. Hence, the algorithm is only assured to find a local minimum in the cost landscape. We find evidence for multiple local minima (for an example, see Supplementary Section 5), but the results presented in the following are minimum-cost solutions that were repeatedly obtained with different initializations and gradient descent parameters. We will denote the control solutions therefore as the “OC”. However, we want to emphasize that there is no way to guarantee that the global minimum was found.

#### 2.4.8 Translational invariance

If a control signal initiates a transition between two stable states, we expect the success of the transition to be invariant under shifts in time, since earlier or later control signals would lead to the same state transition. If the initial state is a stationary state (see Section 3.2.1, up-to-oscillation task), time shifts can be continuous. If the initial state is an oscillatory state (see Section 3.2.1, oscillation-to-up-task, and Section 3.2.2), time shifts can only be a multiple of the oscillation period. By shifting control signals back in time, we might find solutions with smaller *F*_F_, *F*_cc_, or *F*_var_, since an earlier transition implies a longer transition time before inaccuracy is penalized, while the control cost *F*_E_ remains unchanged. The results presented for the state switch in the mean-field EIF model (Section 3.2.2) are obtained by shifting the originally obtained control signal back in time. A repeated optimization does not change the results.

#### 2.4.9 Computational complexity

Table 2 summarizes the computational complexity for computing the cost functional and its derivative computation:

- The complexity for computing the synchrony and oscillation Fourier cost computation scales linearly with the number *N*_*T*_ = *T*/*dt* of total integration time steps and with the number *N* of network nodes. The computation of its derivative scales quadratically with *N*_*T*_ and linearly with *N*.
- The computational complexity for computing the cross-correlation cost scales linearly with *N*_*T*_ and quadratically with *N*. The same holds true for its derivative.
- The computational complexity for computing the variance cost scales linearly with both *N*_*T*_ and *N*. The same holds true for its derivative.

**Table 2:** Computational complexity of cost functional and cost derivative computation.

$F_{\text{F}}^{\text{sync}}\sim N_{T}$	$F_{\text{F}}^{\text{sync}}\sim N$	$\frac{dF_{\text{F}}^{\text{sync}}}{du}\sim N_{T}^{2}$	$\frac{dF_{\text{F}}^{\text{sync}}}{du}\sim N$
$F_{\text{F}}^{\text{osc}}\sim N_{T}$	$F_{\text{F}}^{\text{osc}}\sim N$	$\frac{dF_{\text{F}}^{\text{osc}}}{du}\sim N_{T}^{2}$	$\frac{dF_{\text{F}}^{\text{osc}}}{du}\sim N$
*F*_cc_~*N*_*T*_	$F_{\text{cc}}\sim N^{2}$	$\frac{dF_{\text{cc}}}{du}\sim N_{T}$	$\frac{dF_{\text{cc}}}{du}\sim N^{2}$
*F*_var_~*N*_*T*_	*F*_var_~*N*	$\frac{dF_{\text{var}}}{du}\sim N_{T}$	$\frac{dF_{\text{var}}}{du}\sim N$

For simulations of long time series, the Fourier cost is outperformed in terms of computational costs by the other two cost functionals. Similarly, for large networks, the cross-correlation cost is outperformed by the other two cost functionals.

## 3 Results

### 3.1 Induction of oscillations

We first apply the Fourier cost defined in Equation 7 to induce oscillations in a single-node system from a constant stationary (up or down) state.

**Figure 4:**
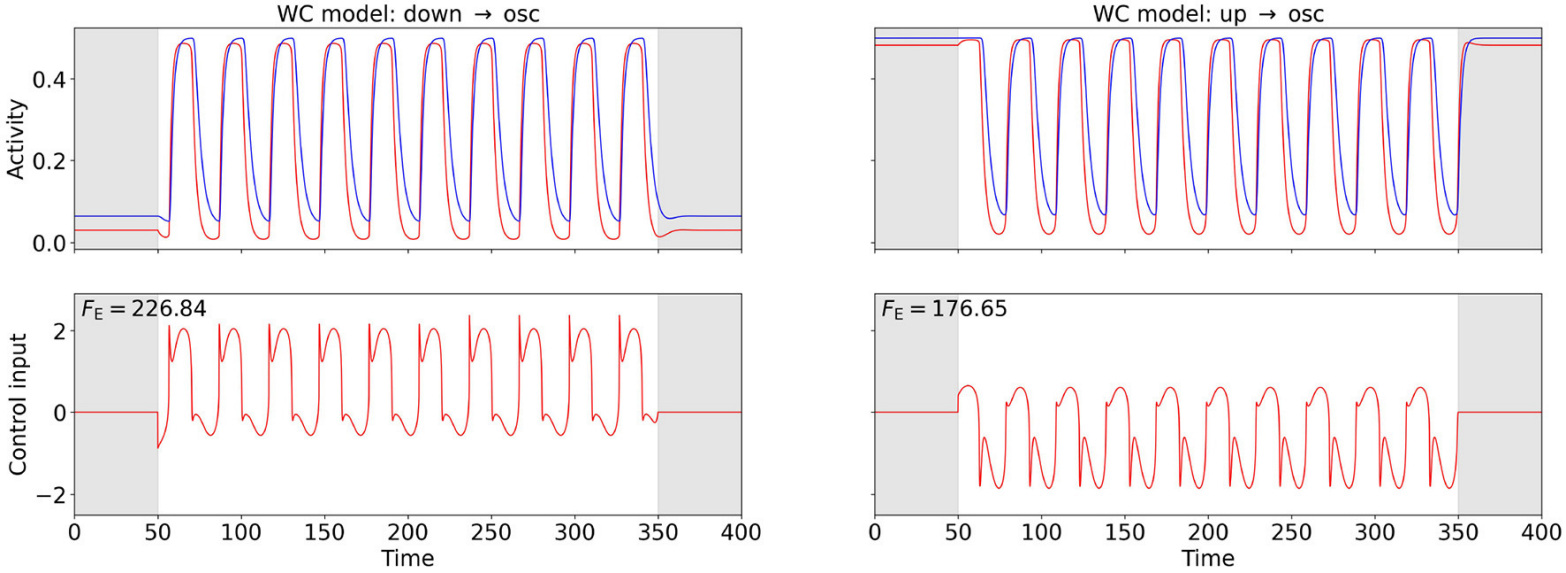
OC using the Fourier cost to induce oscillations at $\tilde{f}=30\text{ Hz}$ from a stationary state in the WC model. The **bottom** row shows the computed OC, and the **top** row shows the resulting time series of the activity variables *E* and *I*. The activity of and the control inputs to the excitatory population are plotted in red, and the activity of the inhibitory population is plotted in blue. The gray-shaded regions (*t* < 50 and *t* > 350) indicate the time intervals during which the control is not active and inaccuracy is not penalized. The energy cost *F*_E_ is provided in the top-left corner of the control plots. The plot on the left-hand side relates to point (A), and the plot on the right-hand side relates to point (B) (cf. Figure 2A). The weight is $w_{\text{F}}=8\cdot 10^{4}$ for both points.

**Figure 5:**
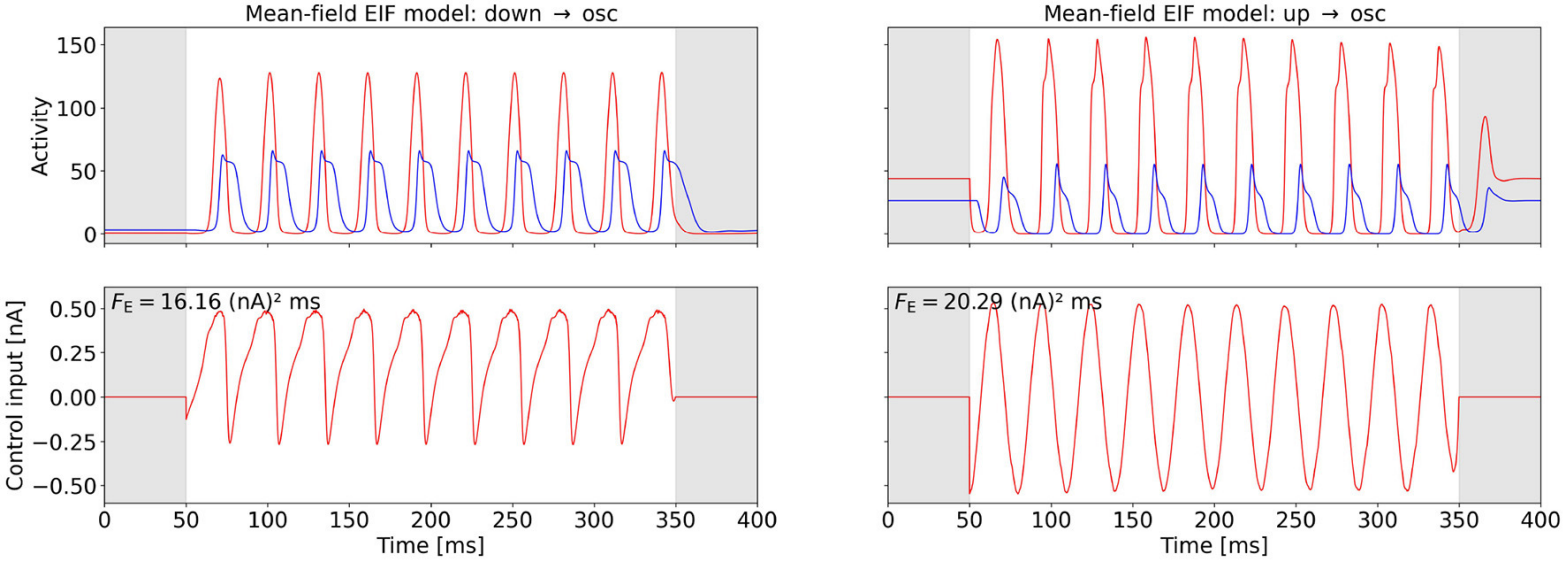
OC using the Fourier cost to induce oscillations at $\tilde{f}=30\text{ Hz}$ from a stationary state in the mean-field EIF model. The **bottom** row shows the computed OC, and the **top** row shows the resulting time series of the activity variables *r*_*E*_ and *r*_*I*_. The activity of and the control inputs to the excitatory population are plotted in red, and the activity of the inhibitory population is plotted in blue. The gray-shaded regions (*t* < 50 ms and *t* > 350 ms) indicate the time intervals during which the control is not active and inaccuracy is not penalized. The energy cost *F*_E_ is provided in the top-left corner of the control plots. The plot on the left-hand side relates to point (A), and the plot on the right-hand side relates to point (B) (cf. Figure 2B). The weight is *w*_F_ = 0.555 for both points. We initialized the OC computation with an oscillatory control signal for point (A).

Figures 4, 5 show the computed OC and the time series of the activity variables, *E* and *I* or *r*_*E*_ and *r*_*I*_, respectively, of the corresponding controlled system at the two points (A) and (B) (cf. Figures 2A, B) for the WC and the mean-field EIF models. The algorithm successfully computes a control input that creates oscillations with the target frequency in all cases. We observe that the shapes of the control signals are comparable for points (A) and (B) within a model but do not transfer across models: For the WC, the periodic OC signals for both tasks have almost vertical slopes and for each period, a broad, secondary peaks follow a sharp, initial peak but in opposite directions, ${\vec{u}}^{\text{down}\to \text{osc}}\approx -{\vec{u}}^{\text{up}\to \text{osc}}$. In comparison, the mean-field EIF OC signals resemble a more or less distorted sine curve. These observations hold true also for other state-space locations within the up- and down-state regimes and for other target frequencies (results not shown).

### 3.2 Switch between stable states

In this Section, we study tasks, in which the control initiates a state switch between coexisting stable states, comparing the effects of the cost functionals *F*_F_, *F*_cc_, and *F*_var_.

#### 3.2.1 Switch between stationary and oscillatory states

The mean-field EIF model exhibits a regime in state space, where a stable state of constant high activity and a stable oscillatory state coexist (see Figure 2B). We apply the Fourier cost to initiate a state switch between these stable states at point (C) in the mean-field EIF state space. For the up-to-oscillations task, we first measure the frequency *f*_C_ of the oscillation at point (C) to then enforce an oscillation at this frequency. For the oscillations-to-up task, we suppress oscillations at frequency *f*_C_ by setting *w*_F_ < 0, thus penalizing this Fourier mode. We compute the minimum-energy control, i.e., the control with the smallest possible *F*_E_ by varying *w*_F_ (see Section 2.4).

**Figure 6:**
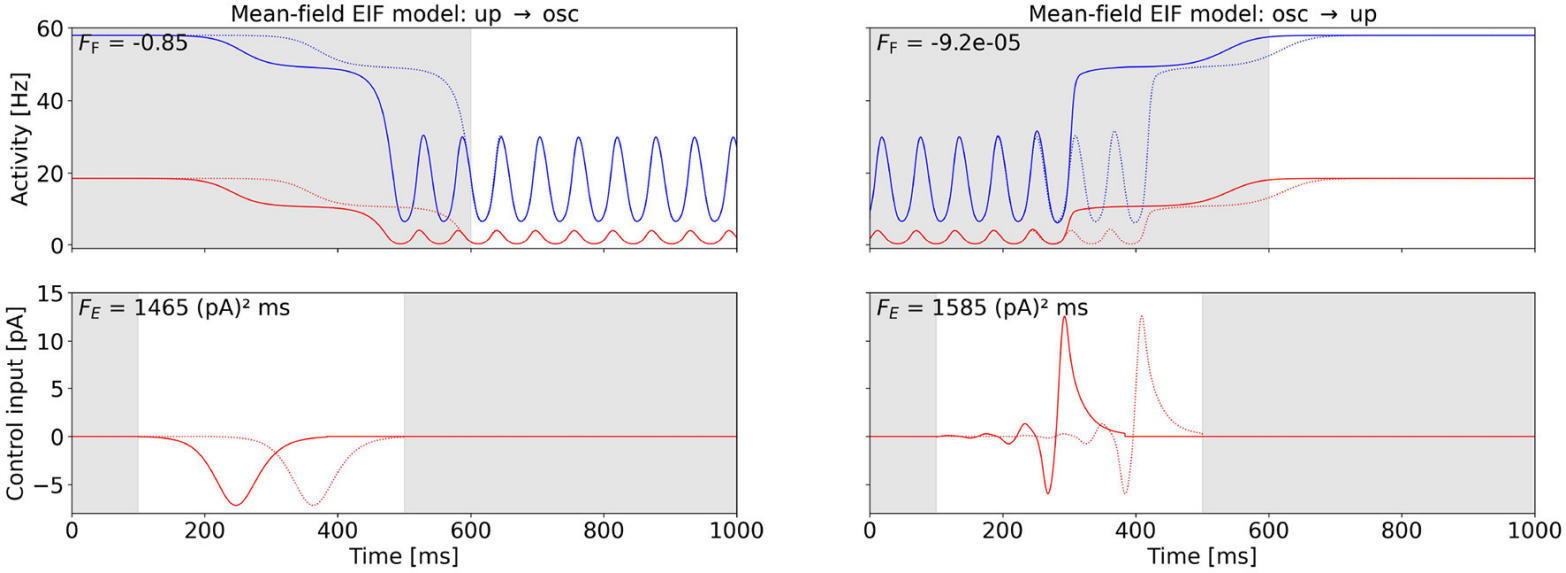
OC to switch between the up state and the oscillatory state in the mean-field EIF model at point (C) (cf. Figure 2B). The **bottom** row shows the computed OC, and the **top** row shows the resulting activity variables *r*_*E*_ and *r*_*I*_, when this control is applied. The activity of and control inputs to the excitatory population are plotted in red, and the activity of the inhibitory population is plotted in blue. The control signals are shifted back in time by two periods (see Section 2.4), and the original control signal and the corresponding original activity are plotted as a dotted line. The shift improves the Fourier cost by 0.67% (**left**) and 99.36% (**right**). The Fourier cost *F*_F_ corresponding to the control shifted back in time (solid line) is provided in the top-left corner of the activity plots. The gray-shaded regions indicate the time intervals during which the control is not active (*t* < 100 ms and *t* > 500 ms) and inaccuracy is not penalized (*t* < 600 ms). The energy cost *F*_E_ is provided in the top-right corner of the control plots. The switch from the up state to the oscillatory state is shown on the left-hand side. Here, we initialize the system in the stationary up state and set the target frequency to *f*_C_. The weight is initialized at *w*_F_ = 1, 000 and decreased to *w*_F_ = 0.0429 (cf. Section 2.4). The switch from the oscillatory state to the up state is shown on the right-hand side. Here, we initialize the system in the oscillatory state and suppress the frequency *F*_C_ with a negative weight initialized with *w*_F_ = −1 and increased to *w*_F_ = −0.0502 (cf. Section 2.4). For this task, we initially enable control for 400 < *t* < 500 and set the control interval as shown after a few iterations. Otherwise, we only find the local OC shown in the Supplementary Section 5.

Figure 6 shows the OC and the corresponding activities of the excitatory and inhibitory populations for the two control tasks. We observe that the state switch is successful for both tasks and that the control signal exhibits a short pulse close to the end of the control interval. The control input is approximately equally strong for both tasks (see Figure 6 for numerical values). For the oscillations-to-up task, *F*_E_ differs by less than 2% when we vary the oscillation phase at which control is activated (results not shown).

#### 3.2.2 Switch between in-phase and out-of-phase oscillation

We consider a symmetric WC two-node network (coupling scheme sketched in Figure 3A) at point (C) in state space (see Figure 2A), where a stable IP oscillation coexists with a stable OOP oscillation. We first apply the OC algorithm using $F_{\text{F}}^{\text{sync}}$, *F*_cc_, and *F*_var_ to compute control signals to switch from OOP and IP. We set the target frequency to $\tilde{f}=f_{\text{IP}}$ for the synchronization Fourier cost. We then use $F_{\text{F}}^{\text{osc}}$, *F*_cc_, and *F*_var_ to switch from IP to OOP. We set the target frequency to $\tilde{f}=f_{\text{OOP}}$ for the oscillation Fourier cost, and chose negative weights *w*_cc_, *w*_var_ < 0 for cross-correlation and variance cost to penalize synchrony. We compute the minimum-energy control, i.e., the control with the smallest possible *F*_E_, by varying *w*_F_ (see Section 2.4). We find that all cost functionals produce the same OC in this limit.

**Figure 7:**
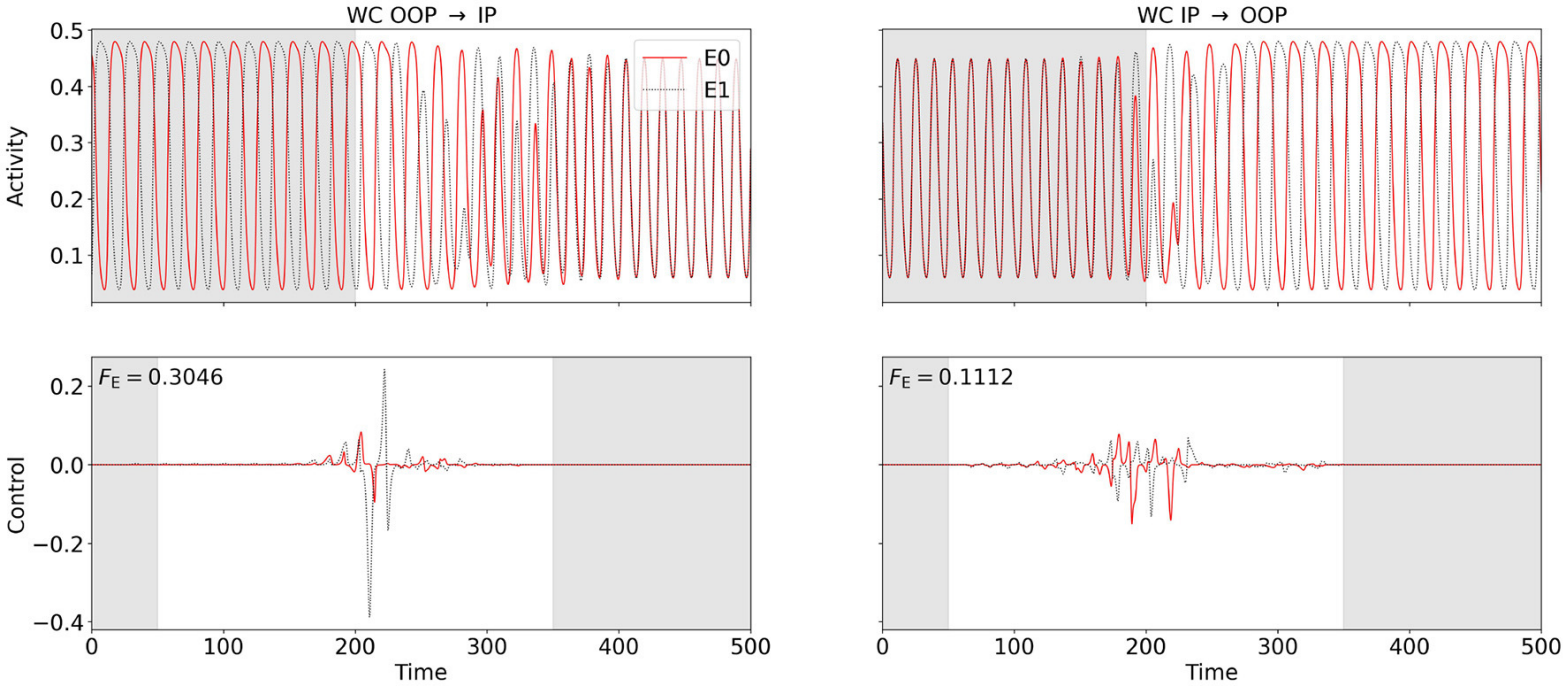
OC switches between in-phase (IP) and out-of-phase (OOP) oscillations in the two-node WC network at point (C) (cf. Figure 2A). We use $F_{\text{F}}^{\text{sync}}$, *F*_cc_, and *F*_var_ for OOP → IP and $F_{\text{F}}^{\text{osc}}$, *F*_cc_, and *F*_var_ for IP → OOP. For any of the cost functionals, the same OC is obtained in the minimum-energy limit. The **bottom** row shows the computed OC, and the **top** row shows the resulting excitatory activity. Excitatory activity of and control inputs to node 0 (node 1) are plotted in solid red (dotted black). The control signals are shifted back in time by two periods (see Section 2.4). Control is enabled between 50 and 350 (disabled in the gray-shaded regions in the control plots), Fourier, cross-correlation, or variance costs are evaluated between 200 and 600 (ignored in the gray-shaded region in the activity plots). The switch from OOP to IP is shown on the left-hand side, and the switch from IP to OOP is shown on the right-hand side. The energy cost *F*_E_ is provided in the top-left corner of the control plots. For the OOP-to-IP switch, the weights are initialized as $w_{\text{F}}^{\text{sync}}=4000$, *w*_cc_ = 250, and *w*_var_ = 30000 and are dynamically reduced to $w_{\text{F}}^{\text{sync}}=17.01$, *w*_cc_ = 0.8361, and *w*_var_ = 12.47. For the IP-to-OOP switch, the weights are initialized as $w_{\text{F}}^{\text{osc}}=2000$, *w*_cc_ = −500, and *w*_var_ = −1000 and are dynamically adjusted to $w_{\text{F}}^{\text{osc}}=7.284$, *w*_cc_ = −2.260, and *w*_var_ = −3.979.

Figure 7 shows the results. For the switch from IP to OOP, the total required energy input *F*_E_ is less than half as strong as for the switch from OOP to IP (see Figure 7 for numerical values). Similarly as in Section 3.2.1, control costs differ only marginally when varying the oscillation phase at which control is activated (results not shown).

### 3.3 (De-) Synchronization of oscillating networks

To study how networks can be synchronized or desynchronized, we consider a six-node WC network (coupling scheme sketched in Figure 3B), for which we find a state of asynchronous oscillation at point (D) and a state of synchronous oscillation at point (E) in state space (see Figure 2A). We apply $F_{\text{F}}^{\text{sync}}$, *F*_cc_, and *F*_var_ with $w_{\text{F}}^{\text{sync}},w_{\text{cc}},w_{\text{var}}>0$ to compute the OC to synchronize (at point (D)). To desynchronize (at point (E)) the activity of the network, we set $w_{\text{F}}^{\text{sync}},w_{\text{cc}},w_{\text{var}}<0$. We evaluate the Fourier spectrum of the network activity and use its peak frequency as $\tilde{f}$ for the Fourier cost.

**Figure 8:**
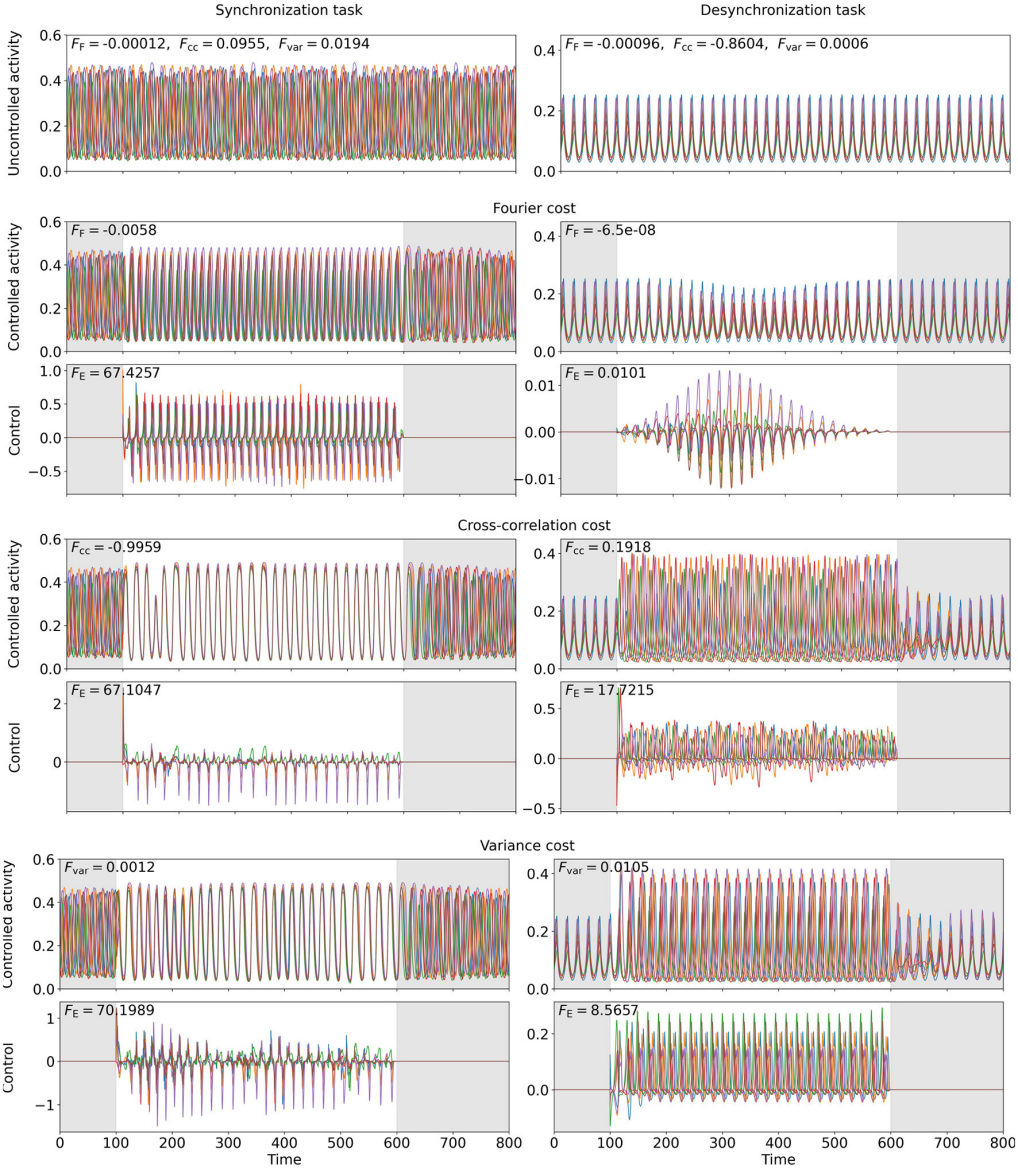
OC to enforce synchrony (**left**, point (D)) or asynchrony (**right**, point (E)) in a six-node WC network. The six colors represent one network node each. The **top** row shows the uncontrolled excitatory activity. Row three (five, seven) shows the computed OC, when the Fourier cost (cross-correlation cost, variance cost) is applied, and row two (four, six) the resulting excitatory activity, if this control is applied. The gray-shaded regions indicate the time intervals during which control is not active and inaccuracy is not penalized (*t* < 100 and *t* > 600). The energy costs *F*_E_ are provided in the top-left corners of the control plots. $F_{\text{F}}^{\text{sync}}$, *F*_cc_, and *F*_var_ are provided in the top-left corner of the activity plots. The weights are $w_{\text{F}}^{\text{sync}}=18600$, *w*_cc_ = 4711, and *w*_var_ = 45000 (left) and $w_{\text{F}}^{\text{sync}}=1000$, *w*_cc_ = 500, and *w*_var_ = 2000 (right).

The results are shown in Figure 8. We observe that, while all three cost functionals succeed to synchronize the network at point (D), neither the synchronization Fourier cost nor the variance cost can drive the system into an asynchronous state, even though numerically, the cost contributions $F_{\text{F}}^{\text{sync}}$ and *F*_var_ lead to considerably smaller total costs. For the Fourier-controlled scenario, we analyze the spectrum of the controlled activity and find that the control slightly increases the frequency. Hence, the Fourier component of the original target frequency drops almost to zero. For the variance-controlled scenario, the control shifts the phases slightly, such that the activity of each node deviates from the network mean, while increasing the oscillation amplitude, such that the difference between node activity and mean increases. This results in a considerable increase in *F*_var_.

We compute the temporal mean of the Kuramoto order parameter (Acebrón et al., 2005) of the network activity to quantitatively compare the performance of the cost functionals (equations are provided in the Supplementary Section 6). The Kuramoto order parameter ranges from 0 (no synchronization) to 1 (full synchronization). The numerical values are provided in Table 3. All cost functionals succeed to improve the Kuramoto order parameter compared to the uncontrolled activity. In agreement with our observations from the activity plots (see Figure 8), the Fourier cost performs worst, and the cross-correlation cost performs best in terms of increasing (synchronization task) or decreasing (desynchronization task) the Kuramoto order parameter.

**Table 3:** Temporal mean Kuramoto order parameter for the uncontrolled and controlled network activity for the control tasks shown in Figure 8.

**Cost functional**	**Synchronization task**	**Desynchronization task**
uncontrolled	0.32	0.81
$F_{\text{F}}^{\text{sync}}$	0.63	0.73
*F*_cc_	0.99	0.33
*F*_var_	0.94	0.47

The values were computed for the period during which the control was active.

## 4 Discussion

We introduce novel cost functionals that enable to use OCT to induce oscillations or synchrony in non-linear dynamical systems without specifying a reference trajectory. We apply the cost functionals *F*_F_, *F*_cc_, and *F*_var_, to two models of neural population dynamics to study different control tasks. We first enforce oscillations from stationary states using *F*_F_ (see Section 3.1) and observe that OC signals drive oscillations at the given target frequency for all investigated paradigms but that the shapes of the control signals are not transferable across models. Next, we study how one can switch between stable states using *F*_F_, *F*_cc_, and *F*_var_ (see Section 3.2). First, we show that applying *F*_F_, one obtains OC signals that switch between a stable up state and a stable oscillatory state in a single-node mean-field EIF system (see Section 3.2.1). Second, we show that all three cost functionals can produce control signals that switch between stable states of IP and OOP oscillations in a two-node WC network. We observe that all cost functionals lead to the same OC in the minimum-energy limit. Moreover, we use the cost functionals *F*_F_, *F*_cc_, and *F*_var_, to (de-) synchronize larger WC networks (see Section 3.3). We study an asynchronously oscillating six-node WC network and try to synchronize its activity using *F*_F_, *F*_cc_, and *F*_var_ and obtain effective control signals for all cost functionals. Finally, we study a synchronously oscillating six-node WC network and try to desynchronize its activity. Here, only the cross-correlation cost functional creates a control signal that effectively desynchronizes the network activity. Both Fourier and variance costs improve numerically, but do so by either shifting the oscillation frequency or by increasing the oscillation amplitude. In summary, we find that the Fourier cost is well suited to drive oscillations at a certain frequency. For the state-switching tasks, all three cost functionals produce satisfactory results. The same holds true for the synchronization tasks in the six-node network. For the desynchronization task, only the cross-correlation cost performs well.

Our results prove that the suggested methods enable to include oscillatory phenomena into the framework of non-linear OCT beyond (de-) synchronization of coupled phase oscillators. However, the list of cost functionals provided in this study is not exhaustive. In particular, we have evaluated further cost functionals within the framework of the adjoint method and observed inferior performances for at least one control task. A list of these discarded cost functionals is given in the Supplementary Section 7. In addition, we do not present a one-size-fits-all solution but a toolbox of methods. Depending on the control task, one needs to chose adequately from this toolbox, and one might have to make educated guesses for initializations, and on the optimization schedule (i.e., weight choices and changes, changes of the control interval or measurement interval, or shifts of the control signals). We hope that the presented examples help to pick appropriately from the toolbox and tailor the choice for the respective task. Future research may evaluate the applicability of our suggested cost functionals in other optimization schemes.

Physical neural systems are often noisy. This property is not covered here; however, the adjoint method of OCT can be extended to enable the computation of OC in noisy systems (see, e.g., Chouzouris et al., 2021). In this case, the cost functional is defined as the expected value,


(18) $$\begin{array}{l} F\to E\left(F\right)=\underset{M\to \infty }{\lim}\frac{1}{M}\overset{M}{\underset{m=1}{\sum }}F. \end{array}$$

In numerical computations, the expected value is replaced by the mean value over a reasonably large number of realizations *M*,


(19) $$\begin{array}{l} E\left(F\right)\approx \frac{1}{M}\overset{M}{\underset{m=1}{\sum }}F,\;M\in \mathbb{N},M<\infty . \end{array}$$

This affects the computation of the adjoint state and the gradient. Preliminary studies suggest that reliable results can be obtained for the presented cost functionals also in noisy systems. The OC framework available within neurolib (Cakan et al., 2023) includes modules for OC computations in a noisy environment.

One apparent limitation of the presented Fourier cost method is that it requires to define a target oscillation frequency. Otherwise, the Fourier cost can neither drive oscillations nor synchronize a system. However, as peaks in Fourier spectra are the broader, the shorter the simulation duration (see Section 2.4), the requirement for a very precise determination of a target frequency becomes less strict. To drive synchronization, this limitation can be overcome by using other cost functionals.

Furthermore, the presented cross-correlation and variance costs do only enable phase synchrony but not frequency synchrony with two or more network nodes oscillating with fixed phase shifts. The oscillation Fourier cost $F_{\text{F}}^{\text{osc}}$ is partially able to capture such cases as it can induce phase-locked oscillations in a network.

The presented methods could improve our understanding of the internal communication of neural circuits and offer new approaches to design brain stimulation protocols. Natural selection led to energy efficiency in neural communication (Quintela-López et al., 2022), and optimality principles enforced by OCT result in biologically plausible communication strategies. Hence, theoretical studies could enable conclusions on, for example, the role of single populations in controlling oscillatory patterns in the brain. Our framework might also enter research on neurological diseases in which synchrony and asynchrony play crucial roles (see, e.g., Jiruska et al., 2012; Sobayo et al., 2020; Uhlhaas and Singer, 2010; Nimmrich et al., 2015). OCT might improve therapeutic brain stimulation, as specific oscillatory brain dynamics can be targeted optimally, simultaneously reducing unintended side effects.

## Data Availability

The datasets presented in this study can be found in online repositories. The names of the repository/repositories and accession number(s) can be found below: https://github.com/lenasal/neurolib/tree/OC_osc_sync.

## Footnotes

^1^If network nodes oscillate entirely anti-phase, the Fourier components cancel out, and an increase in power will not improve the synchronization Fourier cost.
