# Supplementary material for: A framework for optimal control of oscillations and synchrony applied to non-linear models of neural population dynamics
Source: Front Comput Neurosci. 2024 Dec 6;18:1483100. doi: 10.3389/fncom.2024.1483100 (PMC11658993; doi:10.3389/fncom.2024.1483100)
Supplement: Supplementary file 1 [file Data_Sheet_1.PDF]

frontiers

Supplementary Material

A framework for optimal control of oscillations and synchrony applied to nonlinear models of neural population dynamics

1 THE ADJOINT METHOD

We consider a (nonlinear) dynamical systems

$$\vec{h}(\vec{x}(t), \dot{\vec{x}}(t), \vec{x}_{d_1}, \vec{x}_{d_2}, ..., \vec{x}_{d_N}, \vec{u}(t)) = 0, \tag{S1}$$

with the state vector $\vec{x}$, its time derivative $\dot{\vec{x}}$, and N_d potentially different delays d_i. We use the short-hand notation $\vec{x}_{d_i} = \vec{x}(t - d_i)$. In this study, we consider network systems with N network nodes and a finite simulation time. The state vector $\vec{x}$ contains the stacked state vectors $\vec{x}_n, n \in \{1, 2, ...N\}$, of all nodes. Similarly, $\vec{h}$ contains the stacked system dynamics of all network nodes. We denote the dimensionality of the state vector of a single node as N_x, such that $\{\vec{x}, \vec{h}\} \in \mathbb{R}^{N \cdot N_x}$. $\vec{u}(t)$ is the control input, which may effect one or more components of $\vec{x}_n$ for each node in a network. We assume that the initial conditions $\vec{x}(t) = \vec{X}_0(t), t \leq 0$, are given for the time interval $[-\max d_i, 0]$.

The optimal control $\vec{u}^*(t)$ minimizes the cost functional F,

$$\vec{u}^* = \arg\min_{\vec{u}} F. \tag{S2}$$

Conventionally, the cost functional F can be written as an integral over time,

$$F = \int_0^T f(\vec{x}, \vec{u}) \, dt, \tag{S3}$$

where $[0, T]$ is the considered time interval. In this study, however, this is not generally possible. Therefore, we split our cost functionals into two summands: The first summand depends on the control vector $\vec{u}(t)$ and can be written as an integral

$$F_\text{E} = F_\text{E}(\vec{u}) = \int_0^T f_\text{E}(\vec{u}) \, dt. \tag{S4}$$

The second summand depends on the state vector $\vec{x}$ and cannot in general be written in integral form. We denote it by F_X in the following.

We introduce the adjoint state $\vec{\lambda}(t) \in \mathbb{R}^{N \cdot N_x}$, which functions as a Lagrange multiplier, and consider the Lagrange function

$$\begin{aligned} \mathcal{L} &= F_\text{X} + F_\text{E} + \int_0^T \vec{\lambda}^T \vec{h}(\vec{x}(t), \dot{\vec{x}}(t), \vec{x}_{d_1}, \vec{x}_{d_2}, ..., \vec{x}_{d_N}, \vec{u}(t)) \, dt \\ &= F_\text{X}(\vec{x}(t)) + \int_0^T f_\text{E}(\vec{u}(t)) + \vec{\lambda}^T \vec{h}(\vec{x}(t), \dot{\vec{x}}(t), \vec{x}_{d_1}, \vec{x}_{d_2}, ..., \vec{x}_{d_N}, \vec{u}(t)) \, dt. \end{aligned} \tag{S5}$$

The derivative with respect to the control is

$$\frac{d}{d\vec{u}}\mathcal{L} = \frac{d}{d\vec{u}}F = \frac{dF_{\mathrm{X}}(\vec{x})}{d\vec{u}} + \int_0^T \frac{\partial f_{\mathrm{E}}}{\partial \vec{u}} + \vec{\lambda}^T \left(\frac{\partial \vec{h}}{\partial \vec{x}}\frac{d\vec{x}}{d\vec{u}} + \sum_{i=1}^{N_d} \underbrace{\frac{\partial \vec{h}}{\partial \vec{x}_{d_i}}\frac{d\vec{x}_{d_i}}{d\vec{u}}}_{(1)} + \underbrace{\frac{\partial \vec{h}}{\partial \dot{\vec{x}}}\frac{d\dot{\vec{x}}}{d\vec{u}}}_{(2)} + \frac{\partial \vec{h}}{\partial \vec{u}} \right) dt. \tag{S6}$$

The integrand contains vectors $\{\frac{\partial f_{\mathrm{E}}}{\partial \vec{u}}, \vec{\lambda}\} \in \mathbb{R}^{N\cdot N_x}$, and matrices $\{\frac{d\vec{x}}{d\vec{u}}, \frac{d\vec{x}_{d_i}}{d\vec{u}}, \frac{d\dot{\vec{x}}}{d\vec{u}}, \frac{\partial \vec{h}}{\partial \vec{x}}, \frac{\partial \vec{h}}{\partial \vec{x}_{d_i}}, \frac{\partial \vec{h}}{\partial \dot{\vec{x}}}, \frac{\partial \vec{h}}{\partial \vec{u}}\} \in \mathbb{R}^{(N\cdot N_x)\times(N\cdot N_x)}$. Derivatives are defined as $\left(\frac{\partial f_{\mathrm{E}}}{\partial \vec{u}}\right)_i = \frac{\partial f_{\mathrm{E}}}{\partial u_i}$ and $\left(\frac{\partial \vec{h}}{\partial \dot{\vec{x}}}\right)_{ij} = \frac{\partial h_i}{\partial \dot{x}_j}$, etc, where the indices i and j denote the ith and jth component of the respective vector.

A contributions with delayed variables (1) to Equation S6 can be written as

$$\begin{aligned}\int_0^T \vec{\lambda}^T(t) \frac{\partial \vec{h}}{\partial \vec{x}_{d_i}}\bigg|_t \frac{d\vec{x}_{d_i}}{d\vec{u}}\bigg|_t dt &= \int_{-d_i}^{T-d_i} \vec{\lambda}^T(t+d_i) \frac{\partial \vec{h}}{\partial \vec{x}_{d_i}}\bigg|_{t+d_i} \frac{d\vec{x}}{d\vec{u}}\bigg|_t dt \\ &= \int_0^T \chi_{[0,T-d_i]} \vec{\lambda}^T(t+d_i) \frac{\partial \vec{h}}{\partial \vec{x}_{d_i}}\bigg|_{t+d_i} \frac{d\vec{x}}{d\vec{u}}\bigg|_t dt\end{aligned} \tag{S7}$$

where we have used that $\frac{d\vec{x}}{d\vec{u}}\big|_{t\leq 0} = 0$ as the initial condition $\vec{X}_0(t)$ does not depend on the control. $\chi_{[t_a,t_b]}$ denotes the indicator function of the time interval $[t_a, t_b]$.

For the contribution (2) to Equation S6, we can perform a partial integration

$$\int_0^T \left(\vec{\lambda}^T \frac{\partial \vec{h}}{\partial \dot{\vec{x}}}\right) \frac{d\dot{\vec{x}}}{d\vec{u}} dt = \left[\vec{\lambda}^T \frac{\partial \vec{h}}{\partial \dot{\vec{x}}}\frac{d\vec{x}}{d\vec{u}}\right]_0^T - \int_0^T \frac{d}{dt}\left(\vec{\lambda}^T \frac{\partial \vec{h}}{\partial \dot{\vec{x}}}\right) \frac{d\vec{x}}{d\vec{u}} dt = -\int_0^T \left(\dot{\vec{\lambda}}^T \frac{\partial \vec{h}}{\partial \dot{\vec{x}}} + \vec{\lambda}^T \frac{d}{dt}\frac{\partial \vec{h}}{\partial \dot{\vec{x}}}\right) \frac{d\vec{x}}{d\vec{u}} dt. \tag{S8}$$

We require that $\lambda(T) = 0$ and observe that $\frac{d\vec{x}}{d\vec{u}}|_{t=0} = 0$, such that the boundary terms vanish.

We will show in Section 2 that for all our choices of F_{X}, it is possible to write $\frac{dF_{\mathrm{X}}(\vec{x})}{d\vec{u}}$ in the form

$$\frac{dF_{\mathrm{X}}(\vec{x})}{d\vec{u}} = \int_0^T \vec{g}_{\mathrm{X}}(\vec{x}) \frac{d\vec{x}}{d\vec{u}} dt \tag{S9}$$

for some function $g_{\mathrm{X}}(\vec{x})$. Hence, we can write the derivative of the cost as

$$\begin{aligned}\frac{d}{d\vec{u}}F = \int_0^T \frac{\partial f_{\mathrm{E}}}{\partial \vec{u}} + \vec{\lambda}^T \frac{\partial \vec{h}}{\partial \vec{u}} + \Bigg\{ &\vec{g}_{\mathrm{X}}(\vec{x}) + \vec{\lambda}^T(t) \frac{\partial \vec{h}}{\partial \vec{x}} + \sum_{i=1}^{N_d} \chi_{[0,T-d_i]} \vec{\lambda}^T(t+d_i) \frac{\partial \vec{h}}{\partial \vec{x}_{d_i}}\bigg|_{t-d_i} \\ &-\vec{\lambda}^T \frac{d}{dt}\frac{\partial \vec{h}}{\partial \dot{\vec{x}}} - \dot{\vec{\lambda}}(t)^T \frac{\partial \vec{h}}{\partial \dot{\vec{x}}} \Bigg\} \frac{d\vec{x}}{d\vec{u}} dt.\end{aligned} \tag{S10}$$

As the derivative $\frac{d\vec{x}}{d\vec{u}}$ of the state vector with respect to the control is difficult to compute, we require that the term in curly brackets vanishes,

$$\dot{\vec{\lambda}}(t)^T \frac{\partial \vec{h}(t)}{\partial \dot{\vec{x}}} = \vec{g}_{\mathrm{X}}(\vec{x}) + \vec{\lambda}^T(t) \frac{\partial \vec{h}(t)}{\partial \vec{x}} + \sum_i \chi_{[0,T-d_i]} \vec{\lambda}^T(t+d_i) \frac{\partial \vec{h}(t+d_i)}{\partial \vec{x}} - \vec{\lambda}^T \frac{d}{dt}\frac{\partial \vec{h}}{\partial \dot{\vec{x}}}. \tag{S11}$$

Together with the boundary condition $\vec{\lambda}(T) = 0$, this defines the adjoint state, which can be computed by backward integration. The derivative of the cost functional is then given by

$$\frac{d}{d\vec{u}}F = \int_0^T \frac{\partial f_{\text{E}}}{\partial \vec{u}} + \vec{\lambda}^T \frac{\partial \vec{h}}{\partial \vec{u}}\, dt. \tag{S12}$$

In the dynamical systems studied in this work, $\frac{\partial \vec{h}}{\partial \dot{\vec{x}}}$ is a constant, such that $\frac{d}{dt}\frac{\partial \vec{h}}{\partial \dot{\vec{x}}} = 0$. The adjoint Equation S11 simplifies to

$$\dot{\vec{\lambda}}(t)^T \frac{\partial \vec{h}(t)}{\partial \dot{\vec{x}}} = \vec{g}_{\text{X}}(\vec{x}) + \vec{\lambda}^T(t)\frac{\partial \vec{h}(t)}{\partial \vec{x}} + \sum_i \chi_{[0,T-d_i]}\vec{\lambda}^T(t+d_i)\frac{\partial \vec{h}(t+d_i)}{\partial \vec{x}}. \tag{S13}$$

Conventionally, the system equations are formulated such that $\frac{\partial \vec{h}(t)}{\partial \dot{\vec{x}}}$ is a diagonal matrix and $\frac{\partial h_i}{\partial \dot{x}_i} \in \{0, 1\}$. In the WC model, $\frac{\partial \vec{h}(t)}{\partial \dot{\vec{x}}} = \mathbb{1}$, and Equation S13 is a system of delay ODEs. In the mean-field EIF model, the diagonal of $\frac{\partial \vec{h}}{\partial \dot{\vec{x}}}$ also contains zero entries. Hence, Equation S13 is a system of delay differential-algebraic equations (DDAEs). We refer to the supplementary material provided for Salfenmoser and Obermayer (2022) for a detailed description of the adjoint method for DDAEs.

2 DERIVATION OF GRADIENTS OF COST FUNCTIONALS

For the cost functionals that measure oscillation and synchronization, we consider only one relevant state variable for each network node. The formalism we present is, however, not generally limited to this simplification and can be extended straightforwardly. We drop the vector arrow and denote the observable component of the state variable by $x_n(t)$ for network node n.

2.1 The synchronization Fourier cost gradient

The synchronization Fourier cost is defined as (see Section 2.2 in the main article)

$$F_{\text{F}}^{\text{sync}} = -\frac{1}{N^2(T-t_0)^2}\left| \int_{t_0}^T \left(\sum_{n=1}^N x_n(t) \right) \cdot e^{-2\pi i t \tilde{f}}\, dt \right|^2. \tag{S14}$$

In the following, we will use the short-hand notation $\omega = 2\pi\tilde{f}$ and $\Sigma_x(t) = \sum_{n=1}^{N} x_n(t)$. To obtain the derivative $\frac{\partial f_{\mathrm{F}}^{\mathrm{sync}}}{\partial x_n}$ of the synchronization Fourier cost, we first reformulate Equation S14,

$$
\begin{aligned}
F_{\mathrm{F}}^{\mathrm{sync}} &= -\frac{1}{N^2(T-t_0)^2}\left|\int_{t_0}^{T} \Sigma_x(t)\cdot e^{-i\omega t}\, dt\right|^2 \\
&= -\frac{1}{N^2(T-t_0)^2}\left|\int_{t_0}^{T} \Sigma_x(t)\cdot (\cos(-\omega t) + i\sin(-\omega t))\, dt\right|^2 \\
&= -\frac{1}{N^2(T-t_0)^2}\left(\left(\int_{t_0}^{T} \Sigma_x(t)\cdot \cos(-\omega t)\, dt\right)^2 + \left(\int_{t_0}^{T} \Sigma_x(t)\cdot \sin(-\omega t)\, dt\right)^2\right) \\
&= -\frac{1}{N^2(T-t_0)^2}\int_{t_0}^{T}\int_{t_0}^{T} \Sigma_x(t)\Sigma_x(t')\cdot\big(\cos(-\omega t)\cos(-\omega t') + \sin(-\omega t)\sin(-\omega t')\big)\; dt\, dt' \\
&= -\frac{1}{N^2(T-t_0)^2}\int_{t_0}^{T}\int_{t_0}^{T} \Sigma_x(t)\Sigma_x(t')\cdot \cos(\omega(t-t'))\, dt\, dt'.
\end{aligned}
\tag{S15}
$$

We introduce the short-hand notation $C = -\frac{1}{N^2(T-t_0)^2}$ and first compute the derivative $\frac{d\Sigma_x(t)}{d\vec{u}}$,

$$
\frac{d\Sigma_x(t)}{d\vec{u}} = \sum_{k=1}^{N}\frac{\partial\Sigma_x(t)}{\partial x_k}\frac{dx_k}{d\vec{u}} = \sum_{k=1}^{N}\sum_{n=1}^{N}\frac{\partial x_n(t)}{\partial x_k(t)}\frac{dx_k(t)}{d\vec{u}} = \sum_{k=1}^{N}\sum_{n=1}^{N}\delta_{nk}\frac{dx_k(t)}{d\vec{u}} = \sum_{k=1}^{N}\frac{dx_k(t)}{d\vec{u}}. \tag{S16}
$$

It follows that

$$
\begin{aligned}
\frac{dF_{\mathrm{F}}^{\mathrm{sync}}}{d\vec{u}} =& C\int_{t_0}^{T}\int_{t_0}^{T}\frac{d}{d\vec{u}}\left[\Sigma_x(t)\Sigma_x(t')\right]\cdot\cos(\omega(t-t'))\, dt\, dt' \\
=& C\int_{t_0}^{T}\int_{t_0}^{T}\left[\Sigma_x(t')\frac{d\Sigma_x(t)}{d\vec{u}} + \Sigma_x(t)\frac{d\Sigma_x(t')}{d\vec{u}}\right]\cdot\cos(\omega(t-t'))\, dt\, dt' \\
=& C\int_{t_0}^{T}\int_{t_0}^{T}\sum_{k=1}^{N}\left[\Sigma_x(t')\frac{dx_k(t)}{d\vec{u}} + \Sigma_x(t)\frac{dx_k(t')}{d\vec{u}}\right]\cdot\cos(\omega(t-t'))\, dt\, dt' \\
=& \int_{t_0}^{T}\sum_{k=1}^{N}\underbrace{2C\left(\int_{t_0}^{T}\Sigma_x(t')\cos(\omega(t-t'))\, dt'\right)}_{=\left(\vec{g}_{\mathrm{F}}^{\mathrm{sync}}(\vec{x})\right)_k}\frac{dx_k(t)}{d\vec{u}}\, dt \\
=& \int_{t_0}^{T}\sum_{k=1}^{N}\left(\vec{g}_{\mathrm{F}}^{\mathrm{sync}}(\vec{x})\right)_k\frac{dx_k(t)}{d\vec{u}}\, dt = \int_{t_0}^{T}\vec{g}_{\mathrm{F}}^{\mathrm{sync}}(\vec{x})\frac{d\vec{x}}{d\vec{u}}\, dt.
\end{aligned}
\tag{S17}
$$

Hence, we can insert

$$
\left(\vec{g}_{\mathrm{F}}^{\mathrm{sync}}(\vec{x})\right)_k = -\frac{2}{N^2(T-t_0)^2}\int_{t_0}^{T}\Sigma_x(t')\cos(\omega(t-t'))\, dt' \tag{S18}
$$

into the expression for the adjoint state Equation S13.

2.2 The oscillation Fourier cost gradient

The oscillation Fourier cost is defined as (see Section 2.2 in the main article)

$$F_{\mathrm{F}}^{\mathrm{osc}} = -\frac{1}{N(T-t_0)^2} \sum_{n=1}^{N} \left| \int_{t_0}^{T} x_n(t) \cdot e^{-i\omega t} \, dt \right|^2 . \tag{S19}$$

To obtain the derivative $\frac{\partial f_{\mathrm{F}}^{\mathrm{osc}}}{\partial x_n}$ of the oscillation Fourier cost, we first reformulate Equation S19,

$$F_{\mathrm{F}}^{\mathrm{osc}} = -\frac{1}{N(T-t_0)^2} \sum_{n=1}^{N} \int_{t_0}^{T} \int_{t_0}^{T} x_n(t) x_n(t') \cdot \cos(\omega(t-t')) \, dt \, dt' \tag{S20}$$

Similarly as in Section 2.1, we obtain

$$(\vec{g}_{\mathrm{F}}^{\mathrm{osc}}(\vec{x}))_k = -\frac{2}{N(T-t_0)^2} \int_{t_0}^{T} x_k(t') \cdot \cos(\omega(t-t')) \, dt', \tag{S21}$$

which is inserted into the expression for the adjoint state Equation S13.

2.3 The cross-correlation cost gradient

The cross-correlation cost is defined as

$$F_{\mathrm{cc}} = -\int_{t_0}^{T} \frac{2}{N(N-1)(T-t_0)} \sum_{\substack{n=1 \\ l=n+1}}^{N} \frac{(x_n(t)-\overline{x}_n)(x_l(t)-\overline{x}_l)}{\sigma(x_n)\sigma(x_l)} \, dt, \tag{S22}$$

where $\overline{x} = \frac{1}{T-t_0}\int_{t_0}^{T} x(t)\, dt$ is the temporal mean of x, and $\sigma^2(x) = \frac{1}{T-t_0}\int_{t_0}^{T} (x(t)-\overline{x})^2 \, dt$ is its variance. We will use the short-hand notation $C = -\frac{2}{N(N-1)(T-t_0)}$.

We compute the total derivative of the cross-correlation cost functional with respect to the control $\vec{u}$,

$$\begin{aligned} \frac{d}{d\vec{u}} F_{\mathrm{cc}} =& C \int_{t_0}^{T} \frac{d}{d\vec{u}} \sum_{\substack{n=1 \\ l=n+1}}^{N} \frac{(x_n(t)-\overline{x}_n)(x_l(t)-\overline{x}_l)}{\sigma(x_n)\sigma(x_l)} \, dt \\ =& C \int_{t_0}^{T} \sum_{\substack{n=1 \\ l=n+1}}^{N} \left(\frac{1}{\sigma(x_n)\sigma(x_l)} \underbrace{\frac{d}{d\vec{u}} \left[(x_n(t)-\overline{x}_n)(x_l(t)-\overline{x}_l) \right]}_{=(1)} + (x_n(t)-\overline{x}_n)(x_l(t)-\overline{x}_l) \underbrace{\frac{d}{d\vec{u}} \frac{1}{\sigma(x_n)\sigma(x_l)}}_{=(2)} \right) dt \end{aligned} \tag{S23}$$

We consider expression (1):

$$
\begin{aligned}
(1) &= \left((x_l(t) - \overline{x}_l) \frac{d}{d\vec{u}}(x_n(t) - \overline{x}_n) + (x_n(t) - \overline{x}_n) \frac{d}{d\vec{u}}(x_l(t) - \overline{x}_l) \right) \\
&= \left((x_l(t) - \overline{x}_l) \sum_{k=1}^{N} \delta_{nk} \left[\frac{d}{d\vec{u}} x_k(t) - \frac{1}{T - t_0} \int_{t_0}^{T} \frac{d}{d\vec{u}} x_k(t')\, dt' \right] \right. \\
&\quad \left. + (x_n(t) - \overline{x}_n) \sum_{k=1}^{N} \delta_{lk} \left[\frac{d}{d\vec{u}} x_k(t) - \frac{1}{T - t_0} \int_{t_0}^{T} \frac{d}{d\vec{u}} x_k(t')\, dt' \right] \right)
\end{aligned}
\tag{S24}
$$

We observe that the terms $(x_n(t) - \overline{x}_n)\left(\int_{t_0}^{T} \frac{d}{d\vec{u}} x_k(t')\, dt'\right)$ and $(x_l(t) - \overline{x}_l)\left(\int_{t_0}^{T} \frac{d}{d\vec{u}} x_k(t')\, dt'\right)$ vanish after performing the integration in Equation S23 because their second factors no longer depend on time.

We consider expression (2):

$$
\begin{aligned}
(2) &= \left(-\frac{1}{2\sigma(x_l)\sigma^3(x_n)} \frac{d}{d\vec{u}}(\sigma^2(x_n)) - \frac{1}{2\sigma(x_n)\sigma^3(x_l)} \frac{d}{d\vec{u}}(\sigma^2(x_l)) \right) \\
&= -\left(\frac{1}{2\sigma(x_l)\sigma^3(x_n)(T - t_0)} \int_{t_0}^{T} \frac{d}{d\vec{u}} \left[x_n(t) - \frac{1}{T - t_0} \int_{t_0}^{T} x_n(t')\, dt' \right]^2 dt \right. \\
&\quad \left. + \frac{1}{2\sigma(x_n)\sigma^3(x_l)(T - t_0)} \int_{t_0}^{T} \frac{d}{d\vec{u}} \left[x_l(t) - \frac{1}{T - t_0} \int_{t_0}^{T} x_l(t')\, dt' \right]^2 dt \right) \\
&= -\left(\frac{1}{2\sigma(x_l)\sigma^3(x_n)(T - t_0)} \int_{t_0}^{T} \sum_{k=1}^{N} \delta_{nk} 2(x_k(t) - \overline{x}_k) \left[\frac{d}{d\vec{u}} x_k(t) - \frac{1}{T - t_0} \int_{t_0}^{T} \frac{d}{d\vec{u}} x_k(t')\, dt' \right] dt \right. \\
&\quad \left. + \frac{1}{2\sigma(x_n)\sigma^3(x_l)(T - t_0)} \int_{t_0}^{T} \sum_{k=1}^{N} \delta_{lk} 2(x_k(t) - \overline{x}_k) \left[\frac{d}{d\vec{u}} x_k(t) - \frac{1}{T - t_0} \int_{t_0}^{T} \frac{d}{d\vec{u}} x_k(t')\, dt' \right] dt \right)
\end{aligned}
\tag{S25}
$$

Both terms of the form $\int_{t_0}^{T} (x_k(t) - \overline{x}_k) \left[\int_{t_0}^{T} \frac{d}{d\vec{u}} x_k(t')\, dt' \right] dt$ vanish. We then insert the expressions for (1) and (2) without the vanishing terms into $\frac{d}{d\vec{u}} F_{\mathrm{cc}}$ and obtain

$$
\begin{aligned}
\frac{d}{d\vec{u}} F_{\mathrm{cc}} =& C \int_{t_0}^{T} \sum_{\substack{n=1 \\ l=n+1}}^{N} \sum_{k=1}^{N} \left(\frac{1}{\sigma(x_n)\sigma(x_l)} \left(\delta_{nk}(x_l(t) - \overline{x}_l) + \delta_{lk}(x_n(t) - \overline{x}_n) \right) \frac{dx_k(t)}{d\vec{u}} \right. \\
& \left. - \frac{(x_n(t) - \overline{x}_n)(x_l(t) - \overline{x}_l)}{\sigma(x_n)\sigma(x_l)(T - t_0)} \frac{\delta_{nk} + \delta_{lk}}{\sigma^2(x_k)} \int_{t_0}^{T} (x_k(t') - \overline{x}_k) \frac{dx_k(t')}{d\vec{u}}\, dt' \right) dt.
\end{aligned}
\tag{S26}
$$

If $n = k$, then $l \neq k$. Hence, when taking the sum over n, only one contribution of δ_{nk} and δ_{lk} remains. We find

$$\begin{aligned}
\frac{d}{d\vec{u}}F_{\text{cc}} =& C\int_{t_0}^{T}\sum_{\substack{l=1\\l\neq k}}^{N}\sum_{k=1}^{N}\left(\frac{(x_l(t)-\overline{x}_l)}{\sigma(x_k)\sigma(x_l)}\frac{dx_k(t)}{d\vec{u}} - \frac{(x_k(t)-\overline{x}_k)(x_l(t)-\overline{x}_l)}{\sigma(x_k)^3\sigma(x_l)(T-t_0)}\int_{t_0}^{T}(x_k(t')-\overline{x}_k)\frac{dx_k(t')}{d\vec{u}}\,dt'\right)\,dt\\
=& C\sum_{\substack{l=1\\l\neq k}}^{N}\sum_{k=1}^{N}\left(\int_{t_0}^{T}\frac{(x_l(t)-\overline{x}_l)}{\sigma(x_k)\sigma(x_l)}\frac{dx_k(t)}{d\vec{u}}\,dt - \int_{t_0}^{T}\frac{(x_k(t)-\overline{x}_k)(x_l(t)-\overline{x}_l)}{\sigma(x_k)^3\sigma(x_l)(T-t_0)}\,dt\int_{t_0}^{T}(x_k(t')-\overline{x}_k)\frac{dx_k(t')}{d\vec{u}}\,dt'\right)\\
=& C\sum_{\substack{l=1\\l\neq k}}^{N}\sum_{k=1}^{N}\left(\int_{t_0}^{T}\frac{(x_l(t)-\overline{x}_l)}{\sigma(x_k)\sigma(x_l)}\frac{dx_k(t)}{d\vec{u}}\,dt - \int_{t_0}^{T}(x_k(t)-\overline{x}_k)\frac{dx_k(t)}{d\vec{u}}\,dt\left(\int_{t_0}^{T}\frac{(x_k(t')-\overline{x}_k)(x_l(t')-\overline{x}_l)}{\sigma(x_k)^3\sigma(x_l)(T-t_0)}\,dt'\right)\right)\\
=& C\sum_{\substack{l=1\\l\neq k}}^{N}\sum_{k=1}^{N}\left(\int_{t_0}^{T}\left(\frac{(x_l(t)-\overline{x}_l)}{\sigma(x_k)\sigma(x_l)} - \frac{(x_k(t)-\overline{x}_k)}{\sigma(x_k)^3\sigma(x_l)(T-t_0)}\underbrace{\left(\int_{t_0}^{T}(x_k(t')-\overline{x}_k)(x_l(t')-\overline{x}_l)\,dt'\right)}_{=\mathcal{I}_{kl}}\right)\frac{dx_k(t)}{d\vec{u}}\,dt\right)\\
=& \int_{t_0}^{T}\sum_{k=1}^{N}\underbrace{C\sum_{\substack{l=1\\l\neq k}}^{N}\left(\frac{(x_l(t)-\overline{x}_l)}{\sigma(x_k)\sigma(x_l)} - \frac{(x_k(t)-\overline{x}_k)\mathcal{I}_{kl}}{\sigma(x_k)^3\sigma(x_l)(T-t_0)}\right)}_{=(\vec{g}_{\text{cc}}(\vec{x}))_k}\frac{dx_k(t)}{d\vec{u}}\,dt.
\end{aligned} \tag{S27}$$

Hence, we can insert

$$(\vec{g}_{\text{cc}}(\vec{x}))_k = -\frac{2}{N(N-1)(T-t_0)}\sum_{\substack{l=1\\l\neq k}}^{N}\left(\frac{(x_l(t)-\overline{x}_l)}{\sigma(x_k)\sigma(x_l)} - \frac{(x_k(t)-\overline{x}_k)\mathcal{I}_{kl}}{\sigma(x_k)^3\sigma(x_l)(T-t_0)}\right) \tag{S28}$$

into the expression for the adjoint state Equation S13.

2.4 The variance cost gradient

The variance cost is defined as

$$F_{\text{var}} = \frac{1}{N(T-t_0)}\int_{t_0}^{T}\sum_{n=1}^{N}\left(x_n(t)-\overline{x}(t)\right)^2\,dt, \tag{S29}$$

where $\overline{x}(t) = \frac{1}{N}\sum_{n=1}^{N} x_n(t)$ is the network mean of x. The derivative $\frac{dF_{\text{var}}}{d\vec{u}}$ of the variance cost with respect to the control $\vec{u}$ is given by

$$
\begin{aligned}
\frac{dF_{\text{var}}}{d\vec{u}} =& \frac{1}{N(T-t_0)} \int_{t_0}^{T} \sum_{k=1}^{N} \frac{\partial}{\partial x_k} \left(\sum_{n=1}^{N} (x_n(t) - \overline{x}(t))^2 \right) \frac{dx_k}{d\vec{u}}\, dt \\
=& \frac{1}{N(T-t_0)} \int_{t_0}^{T} \sum_{k=1}^{N} \frac{\partial}{\partial x_k} \left(\sum_{\substack{n=1\\ n\neq k}}^{N} (x_n(t) - \overline{x}(t))^2 + (x_k(t) - \overline{x}(t))^2 \right) \frac{dx_k}{d\vec{u}}\, dt \\
=& \frac{1}{N(T-t_0)} \int_{t_0}^{T} \sum_{k=1}^{N} \left(2 \sum_{\substack{n=1\\ n\neq k}}^{N} (x_n(t) - \overline{x}(t))(-\frac{1}{N}) + 2(x_k(t) - \overline{x}(t))(1 - \frac{1}{N}) \right) \frac{dx_k}{d\vec{u}}\, dt \\
=& \frac{1}{N(T-t_0)} \int_{t_0}^{T} \sum_{k=1}^{N} \left(-\frac{2}{N} \underbrace{\sum_{n=1}^{N} (x_n(t) - \overline{x}(t))}_{=0} + \frac{2}{N}(x_k(t) - \overline{x}(t)) + 2(x_k(t) - \overline{x}(t))(1 - \frac{1}{N}) \right) \frac{dx_k}{d\vec{u}}\, dt \\
=& \frac{2}{N(T-t_0)} \int_{t_0}^{T} \sum_{k=1}^{N} (x_k(t) - \overline{x}(t)) \frac{dx_k}{d\vec{u}}\, dt.
\end{aligned}
\tag{S30}
$$

Hence, we can insert

$$
(\vec{g}_{\text{var}}(\vec{x}))_k = \frac{2(x_k(t) - \overline{x}(t))}{N(T-t_0)} \tag{S31}
$$

into the expression for the adjoint state Equation S13.

3 THE MEAN-FIELD EIF MODEL

The system of differential algebraic equations (DAEs) that defines the dynamics of the mean-field EIF model reads (cf. Augustin et al. (2017); Cakan and Obermayer (2020))

$$\begin{pmatrix} r_E(t) - \Phi_r(\mu_E, \sigma_E) \\ r_I(t) - \Phi_r(\mu_I, \sigma_I) \\ \dot{\mu}_E - \frac{1}{\tau_E(t)}(J_{EE}\overline{s}_{EE}(t) + J_{EI}\overline{s}_{EI}(t) + \mu_E^{\text{ext}} - \mu_E(t)) + u_E(t) \\ \dot{\mu}_I - \frac{1}{\tau_I(t)}(J_{IE}\overline{s}_{IE}(t) + J_{II}\overline{s}_{II}(t) + \mu_I^{\text{ext}} - \mu_I(t)) + u_I(t) \\ \sigma_E(t) - (\frac{2J_{EE}^2\sigma_{s,EE}^2(t)\tau_{s,E}\tau_m}{(1+r_{EE}(t))\tau_m+\tau_{s,E}} + \frac{2J_{EI}^2\sigma_{s,EI}^2(t)\tau_{s,I}\tau_m}{(1+r_{EI}(t))\tau_m+\tau_{s,I}} + (\sigma_E^{\text{ext}})^2)^{\frac{1}{2}} \\ \sigma_I(t) - (\frac{2J_{IE}^2\sigma_{s,IE}^2(t)\tau_{s,E}\tau_m}{(1+r_{IE}(t))\tau_m+\tau_{s,E}} + \frac{2J_{II}^2\sigma_{s,II}^2(t)\tau_{s,I}\tau_m}{(1+r_{II}(t))\tau_m+\tau_{s,I}} + (\sigma_I^{\text{ext}})^2)^{\frac{1}{2}} \\ \tau_E(t) - \Phi_\tau(\mu_E, \sigma_E) \\ \tau_I(t) - \Phi_\tau(\mu_I, \sigma_I) \\ \dot{\overline{s}}_{EE} + \frac{\overline{s}_{EE}(t)}{\tau_{s,E}} - (1 - \overline{s}_{EE}(t)) \cdot \frac{r_{EE}(t)}{\tau_{s,E}} \\ \dot{\overline{s}}_{EI} + \frac{\overline{s}_{EI}(t)}{\tau_{s,I}} - (1 - \overline{s}_{EI}(t)) \cdot \frac{r_{EI}(t)}{\tau_{s,I}} \\ \dot{\overline{s}}_{IE} + \frac{\overline{s}_{IE}(t)}{\tau_{s,E}} - (1 - \overline{s}_{IE}(t)) \cdot \frac{r_{IE}(t)}{\tau_{s,E}} \\ \dot{\overline{s}}_{II} + \frac{\overline{s}_{II}(t)}{\tau_{s,I}} - (1 - \overline{s}_{II}(t)) \cdot \frac{r_{II}(t)}{\tau_{s,I}} \\ \dot{\sigma}_{s,EE}^2 - \frac{1}{\tau_{s,E}^2}\left((1 - \overline{s}_{EE}(t))^2 \cdot \rho_{EE}(t) + (\rho_{EE}(t) - 2\tau_{s,E}(r_{EE}(t) + 1)) \cdot \sigma_{s,EE}^2(t)\right) \\ \dot{\sigma}_{s,EI}^2 - \frac{1}{\tau_{s,I}^2}\left((1 - \overline{s}_{EI}(t))^2 \cdot \rho_{EI}(t) + (\rho_{EI}(t) - 2\tau_{s,I}(r_{EI}(t) + 1)) \cdot \sigma_{s,EI}^2(t)\right) \\ \dot{\sigma}_{s,IE}^2 - \frac{1}{\tau_{s,E}^2}\left((1 - \overline{s}_{IE}(t))^2 \cdot \rho_{IE}(t) + (\rho_{IE}(t) - 2\tau_{s,E}(r_{IE}(t) + 1)) \cdot \sigma_{s,IE}^2(t)\right) \\ \dot{\sigma}_{s,II}^2 - \frac{1}{\tau_{s,I}^2}\left((1 - \overline{s}_{II}(t))^2 \cdot \rho_{II}(t) + (\rho_{II}(t) - 2\tau_{s,I}(r_{II}(t) + 1)) \cdot \sigma_{s,II}^2(t)\right) \end{pmatrix} = 0. \tag{S32}$$

The average population activities $r_\alpha(t), \alpha \in \{E, I\}$, are determined by a precomputed transfer function $\Phi_r(\mu_\alpha, \sigma_\alpha)$. $\Phi_r(\mu_\alpha, \sigma_\alpha)$ is a function of the mean membrane current μ_α and its standard deviation σ_α. μ_α decays exponentially with an effective time scale τ_α, which is given by another precomputed transfer function Φ_τ. The sum $\sum_{\beta=E,I} J_{\alpha\beta}\overline{s}_{\alpha\beta}$ of mean synaptic inputs and the background current μ_α^{ext} counteract this decay. The control inputs $u_E(t)$ and $u_I(t)$ directly affect the membrane currents. The variance σ_α of the membrane current is obtained from the variances $\sigma_{s,\alpha\beta}^2$ of the synaptic inputs and the static external variance $\sigma_\alpha^{\text{ext}}$. The mean synaptic activity $\overline{s}_{\alpha\beta}$ decays exponentially with the time constants $\tau_{s,\beta}$ and increases depending on the activity $r_{\alpha\beta}$ received by population α after the time delay d_α from K_β neurons of population β,

$$r_{\alpha\beta}(t) = \frac{c_{\alpha\beta}}{|J_{\alpha\beta}|} K_\beta \tau_{s,\beta} \cdot r_\beta(t - d_\alpha). \tag{S33}$$

The fraction $\frac{c_{\alpha\beta}}{|J_{\alpha\beta}|}$ of the maximum postsynaptic current amplitude of a typical β to α synapse and the maximum synaptic current from β to α downscales the effect of the incoming rate r_β, whereas the synaptic time constant $\tau_{s,\beta}$ strengthens the received activity. The variance $\sigma_{s,\alpha\beta}^2$ of the mean synaptic activity combines the uncertainties of the different contributions to $\overline{s}_{\alpha\beta}$, with

$$\rho_{\alpha\beta}(t) = \frac{c_{\alpha\beta}^2}{J_{\alpha\beta}^2} K_\beta \tau_{s,\beta}^2 \cdot r_\beta(t - d_\alpha). \tag{S34}$$

In network systems, we add the index n to all variables of network node n. The input to node n from all connected nodes in the network enters through $r_{EE,n}(t)$ and $\rho_{EE,n}(t)$,

$$
\begin{aligned}
r_{EE,n}(t) &= \frac{c_{EE}}{J_{EE}} K_E \tau_{s,E} \cdot r_{E,n}(t - d_E) + \frac{c_{\mathrm{gl}}}{J_{EE}} K_{\mathrm{gl}} \tau_{s,E} \sum_{k=1}^{N} C_{nk} \cdot r_{E,k}(t - D_{nk}), \\
\rho_{EE,n}(t) &= \frac{c_{EE}^2}{J_{EE}^2} K_E \tau_{s,E}^2 \cdot r_{E,n}(t - d_E) + \frac{c_{\mathrm{gl}}^2}{J_{EE}^2} K_{\mathrm{gl}} \tau_{s,E}^2 \sum_{k=1}^{N} C_{nk}^2 \cdot r_{E,k}(t - D_{nk}),
\end{aligned}
\tag{S35}
$$

where C and d denote the coupling and delay matrices. All model parameters and their numerical values are given in Table S1.

Table S1. Parameters of the mean-field EI EIF model (cf. Equation S32). Values are taken from Cakan and Obermayer (2020).

Parameter	Description	Numerical value
J_{EE}	maximum synaptic current from E to E	$2.4\,\mathrm{mV\,ms^{-1}}$
J_{EI}	maximum synaptic current from I to E	$-3.3\,\mathrm{mV\,ms^{-1}}$
J_{IE}	maximum synaptic current from E to I	$2.6\,\mathrm{mV\,ms^{-1}}$
J_{II}	maximum synaptic current from I to I	$-1.6\,\mathrm{mV\,ms^{-1}}$
c_{EE}, c_{IE}	maximum AMPA postsynaptic current (PSC) amplitude	$0.3\,\mathrm{mV\,ms^{-1}}$
c_{EI}, c_{II}	maximum GABA PSC amplitude	$0.5\,\mathrm{mV\,ms^{-1}}$
$\tau_{s,E}$	excitatory synaptic time constant	$2\,\mathrm{ms}$
$\tau_{s,I}$	inhibitory synaptic time constant	$5\,\mathrm{ms}$
C	membrane capacitance	$200\,\mathrm{pF}$
g_L	leak conductance	$10\,\mathrm{nS}$
$\tau_m = C/g_L$	membrane time constant	$20\,\mathrm{ms}$
d_E	synaptic delay to excitatory neurons	$4\,\mathrm{ms}$
d_I	synaptic delay to inhibitory neurons	$2\,\mathrm{ms}$
K_E	mean number of excitatory inputs per neuron	800
K_I	mean number of inhibitory inputs per neuron	200
$\sigma_{E,I}^{\mathrm{ext}}$	standard deviation of external input	$1.5\,\mathrm{mV}/\sqrt{\mathrm{ms}}$
K_{gl}	mean number of excitatory inputs per neuron from the network	250

4 FOURIER COST IN LIMITED-TIME SIMULATIONS

The simulation time impacts the sharpness of the peaks in any numerically evaluated Fourier spectrum. The longer the simulation duration, the sharper the peaks (see Figure S1). For tasks, in which we want to switch to a specific oscillatory state (Section 3.2 in the main article), this leads to a tolerance against variations in the frequency $\tilde{f}$ compared to the natural frequency of the target state.

5 LOCAL VS. GLOBAL COST MINIMA

We use gradient descent to reach a minimum of a cost functional. Hence, the algorithm is only assured to find a local minimum in the cost landscape, and we indeed find evidence for multiple local minima. Figure S2 (right) shows an example for such a locally optimal control for the switch from an oscillatory state to an up state in the mean-field EIF model at point (C) (see Figure 2b in the main article), as discussed in Section 3.2.1 in the main article. The global optimum is shown in comparison.

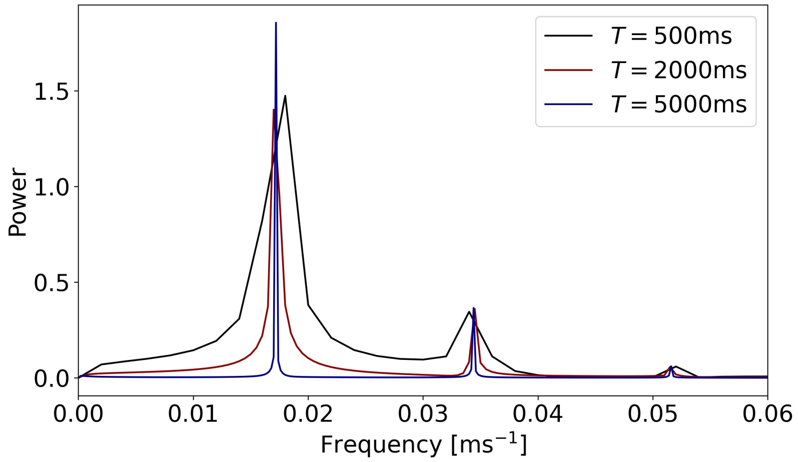

Figure S1. Numerically computed power spectrum of the excitatory activity of the mean-field EIF model in the oscillatory state at point (C) (see Figure 2b in the main article) for different simulation durations.

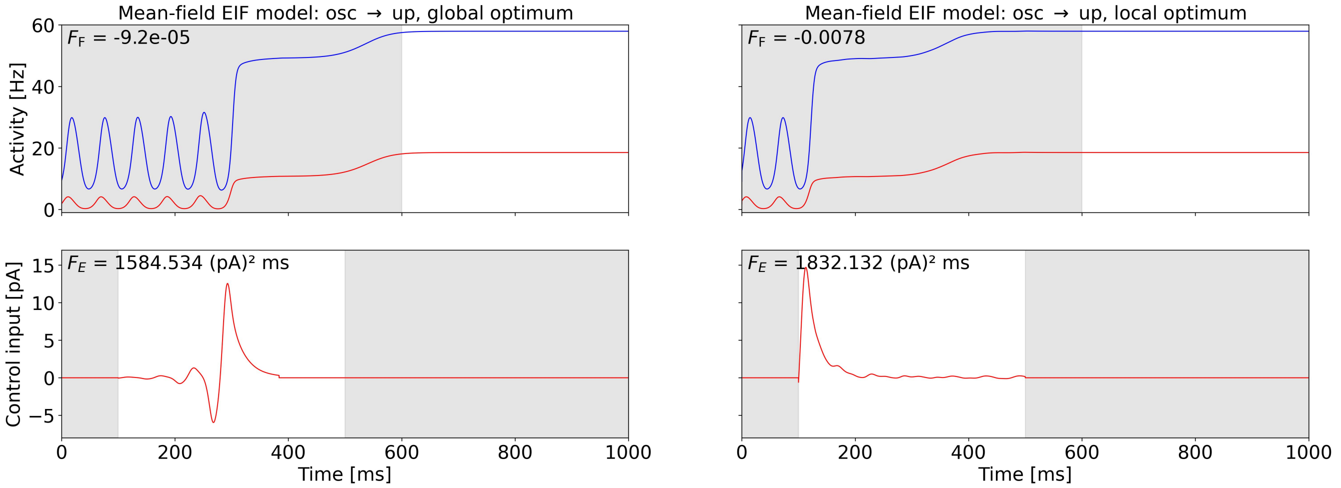

Figure S2. Global and local OC to switch from an oscillatory state to an up state in the mean-field EIF model at point (C) (see Figure 2b in the main article). The bottom row shows the computed control signal, the top row the resulting activity, if this control is applied. The activity of and control inputs to the excitatory population are plotted in red, the activity of the inhibitory population in blue. The gray-shaded regions indicate the time span during which the control is not active ($t < 100\,\mathrm{ms}$ and $t > 500\,\mathrm{ms}$, lower panel) and imprecision is not penalized ($t < 600\,\mathrm{ms}$, upper panel). The globally most energy-efficient control signal is shown on the left-hand side, a local optimal control signal is shown on the right-hand side. The weights are initialized with $w_\mathrm{F} = -1$ and dynamically increased to $w_\mathrm{F} = -0.0498$ (left panel) and $w_\mathrm{F} = -0.0568$ (right panel).

6 THE KURAMOTO ORDER PARAMETER AS A MEASURE OF SYNCHRONIZATION

The Kuramoto order parameter $z(t)$ is a commonly used measure of instantaneous synchronization (Acebrón et al. (2005)). It is defined as

$$z(t) = \frac{1}{N} \sum_{n=1}^{N} e^{i\varphi_n(t)}, \tag{S36}$$

where $\varphi_n(t)$ is the instantaneous phase of the activity $x_n(t)$ of network node n. The instantaneous phase is computed via the Hilbert transform $\hat{x}_n(t)$ of the activity,

$$\varphi_n(t) = \arctan\left(\frac{\hat{x}_n(t)}{x_n(t)}\right). \tag{S37}$$

The Hilbert transform is defined as

$$\hat{x}(t) = \frac{1}{\pi}\,\text{p.v.}\int_{-\infty}^{\infty} \frac{x(t)}{t-t'}\, dt', \tag{S38}$$

where p.v. denotes the Cauchy principal value. The Kuramoto order parameter is a real number that ranges from 0 (no synchronization) to 1 (full synchronization).

The Kuramoto order parameter has been used as a cost functional in studies of OC of synchrony (e.g., Vu et al. (2024)). We can define the synchronization phase cost as

$$F_{\text{phase}}^{\text{sync}} = -\frac{1}{N^2(T-t_0)}\int_{t_0}^{T} \Big|\sum_{n=1}^{N} e^{i\varphi_n(t)}\Big|\, dt, \tag{S39}$$

where $\varphi_n(t)$ is the instantaneous phase of $x_n(t)$. To induce oscillations in a single node, or in individual network nodes, one can define the oscillation phase cost

$$F_{\text{phase}}^{\text{osc}} = -\frac{1}{N(T-\tau-t_0)}\sum_{n=1}^{N}\int_{t_0}^{T-\tau} \left|e^{i\varphi_n(t)} + e^{i\varphi_n(t+\tau)}\right| dt, \tag{S40}$$

where $\tau = \tilde{f}^{-1}$ is the target oscillation period.

These cost functional definitions are, in principle, applicable using the adjoint method. However, F_{K} and $\frac{dF_{\text{K}}}{du}$ scale quadratically and cubically with the number of time steps N_T, respectively. Hence, this cost functional is computationally too expensive to be considered even for control tasks with short simulation duration, when the adjoint method is applied.

7 ALTERNATIVE COST FUNCTIONALS

We explored different ideas for cost functionals that can be included into the adjoint method. The three cost functionals presented in the main article appeared most convincing for our purposes. In this section, we present further options that did not perform as well as others for at least one of the control tasks.

First, we consider cost functionals that do not relate the activity of nodes throughout the network with each other. These cost functionals cannot capture synchrony and are therefore not applicable for any (de-) synchronization task, such as the switch between IP and OOP oscillations (Section 3.2.2 in the main article), and the (de-) synchronization task (Section 3.3 in the main article). The following cost functionals were evaluated for the induction of oscillations (Section 3.1 in the main article) and for the switch between stationary and oscillatory states (Section 3.2.1).

- Autocorrelation cost

$$F_{\text{AC}} = -\frac{1}{N(T-\tau-t_0)} \sum_{n=1}^{N} \int_{t_0}^{T-\tau} (x_n(t) - \overline{x}_n) \cdot (x_n(t+\tau) - \overline{x}_n)\, dt, \tag{S41}$$

where $\tau = \tilde{f}^{-1}$ is the target oscillation period and $\overline{x}_n = \frac{1}{T}\int_{t_0}^{T} x_n(t)$ is the temporal mean of the activity.
Induction of oscillations: F_{AC} is only able to find an efficient control strategy, if the control is initialized with a τ-period signal in a subinterval of $[t_0, T]$. In this case, F_{AC} reproduces the initial control throughout $[t_0, T]$ and reshapes it to become more cost-efficient. This holds true for both models and for both initial conditions (down and up state). However, no oscillations can be created without a periodic initialization of the control signal.
Switch between stationary and oscillatory states: F_{AC} can induce switches between up state and oscillatory state. In the minimum-energy limit, we find the same OC signal as with the oscillation Fourier cost $F_{\text{F}}^{\text{osc}}$.

- Oscillation variance cost

$$F_{\text{var}}^{\text{osc}} = -\frac{1}{N(T-t_0)} \sum_{n=1}^{N} \int_{t_0}^{T} (x_n(t) - \overline{x}_n)^2\, dt, \tag{S42}$$

where $\overline{x}_n = \frac{1}{T}\int_{t_0}^{T} x_n(t)$ is the temporal mean of the activity.
Induction of oscillations: $F_{\text{var}}^{\text{osc}}$ is only able to produce efficient control strategies for oscillations, if initialized with a periodic signal throughout $[t_0, T]$. Any periodic input is reshaped to improve its cost efficiency. However, no oscillations can be created without a periodic initialization of the control signal.
Switch between stationary and oscillatory states: $F_{\text{var}}^{\text{osc}}$ can induce switches between up state and oscillatory state. In the minimum-energy limit, we find the same OC signal as with the oscillation Fourier cost $F_{\text{F}}^{\text{osc}}$.

Second, we consider the mean variance cost (Vu et al. (2024))

$$F_{\text{var}}^{\text{mean}} = -\frac{1}{(T-t_0)} \int_{t_0}^{T} (\overline{x}(t) - \overline{\overline{x}})^2\, dt, \tag{S43}$$

where $\overline{x}(t) = \frac{1}{N}\sum_{n=1}^{N} x_n(t)$ is the network mean and $\overline{\overline{x}} = \frac{1}{N(T-t_0)}\int_{t_0}^{T}\sum_{n=1}^{N} x_n(t)\, dt$ is the temporal mean of the network mean. For $N = 1$, the oscillation variance cost is recovered. The definition of $F_{\text{var}}^{\text{mean}}$ rests on the assumption that only the network mean can be measured, and not the individual node activity. This assumption may be useful for practical applications, in which only the network as a whole can be observed. However, in an analytic scenario, which may be aimed at a more profound mechanistic understanding of network interactions, it may be reasonable to include information on the individual node dynamics, if available. We hypothesize that more detailed information will lead to more efficient control strategies.

We evaluate $F_{\text{var}}^{\text{mean}}$ for the switch between IP and OOP oscillations (Section 3.2.2 in the main article) and for the (de-) synchronization task (Section 3.3 in the main article).
Switch between IP and OOP oscillations: $F_{\text{var}}^{\text{mean}}$ can induce switches between IP and OOP oscillations.

In the minimum-energy limit, we find the same OC signal as with the three cost functionals presented in the main article.

(De-) synchronization of six-node network: $F_{\text{var}}^{\text{mean}}$ successfully improves or impairs network synchrony. We compare the resulting mean Kuramoto order parameters of $F_{\text{var}}^{\text{mean}}$ and F_{var} as defined in the main article: For the synchronization task, we find 0.47 with $F_{\text{var}}^{\text{mean}}$ and 0.94 with F_{var}. For the desynchronization task, we find 0.7 with $F_{\text{var}}^{\text{mean}}$ and 0.47 with F_{var}. Interestingly, $F_{\text{var}}^{\text{mean}}$ decreases the oscillation amplitude for the desynchronization task, while F_{var} increases the oscillation amplitude (see Figure 8 in the main article). According to its definition (see Equation S43), there are two ways to minimize $-F_{\text{var}}^{\text{mean}}$: Either by distributing oscillations such that the average activity remains constant, or by decreasing the oscillation amplitude to (near) zero. Apparently, the latter strategy is more efficient in terms of the total cost. Comparing the mean Kuramoto order parameters, we conclude that F_{var} performs better. We hypothesize that this is due to the larger amount of information that is taken into account for the computation of F_{var} and its gradient.

REFERENCES

Acebrón, J. A., Bonilla, L. L., Pérez Vicente, C. J., Ritort, F., and Spigler, R. (2005). The Kuramoto model: A simple paradigm for synchronization phenomena. *Rev. Mod. Phys.* 77, 137–185. doi:10.1103/RevModPhys.77.137

Augustin, M., Ladenbauer, J., Baumann, F., and Obermayer, K. (2017). Low-dimensional spike rate models derived from networks of adaptive integrate-and-fire neurons: Comparison and implementation. *PLoS Comput Biol* 13, 1–46. doi:10.1371/journal.pcbi.1005545

Cakan, C. and Obermayer, K. (2020). Biophysically grounded mean-field models of neural populations under electrical stimulation. *PLoS Comput Biol* 16, 1–30. doi:10.1371/journal.pcbi.1007822

Salfenmoser, L. and Obermayer, K. (2022). Nonlinear optimal control of a mean-field model of neural population dynamics. *Front Comput Neurosci* 16. doi:10.3389/fncom.2022.931121

Vu, M., Singhal, B., Zeng, S., and Li, J.-S. (2024). Data-driven control of oscillator networks with population-level measurement. *Chaos (Woodbury, N.Y.)* 34. doi:10.1063/5.0191851
